# HistoBreastCAD: an integrated deep learning framework for segmentation and multiclass classification of whole-slide breast histopathology images

**DOI:** 10.3389/fbinf.2026.1870283

**Published:** 2026-10-13

**Authors:** Oswaldo Celi-Vega, Yuliana Jiménez-Gaona, Patricio Corral-Dominguez, Ángel Gutiérrez, Zaida Socola-Barzallo

**Affiliations:** 1 Departamento de Ciencias de La Computación, Universidad Técnica Particular de Loja, Loja, Ecuador; 2 Departamento de Química y Ciencias Exactas, Universidad Técnica Particular de Loja, Loja, Ecuador; 3 Corporación Médica Monte Sinaí-CIPAM (Centro Integral de Patología Mamaria) Cuenca-Ecuador, Facultad de Ciencias Médicas, Universidad de Cuenca, Cuenca, Ecuador; 4 Departamento de Ciencias de La Salud, Universidad Técnica Particular de Loja, Loja, Ecuador

**Keywords:** breast cancer, classification, computer-aided diagnosis, deep learning, histopathology, segmentation, whole-slide imaging

## Abstract

Breast cancer remains a major cause of cancer-related morbidity and mortality worldwide, and histopathological examination remains the diagnostic gold standard. This study presents HistoBreastCAD, an integrated deep learning framework that combines tissue-region segmentation, four-class histopathology classification, spatial mapping of predictions, and interactive whole-slide image (WSI) review. Segmentation was evaluated using WSI-grouped five-fold cross-validation, while classification used source-image-grouped five-fold cross-validation to prevent source-level data leakage. The final DeepLabV3+ L0 segmentation configuration achieved an out-of-fold micro-Dice of **0.8390**, IoU of **0.7226**, precision of **0.8278**, and recall of **0.8505**. For classification, EfficientNetB0 was the strongest individual model, while a frozen equal-weight soft-voting ensemble of EfficientNetB0 and ResNet18-GAP-attention achieved an accuracy of **0.8283 ± 0.0320** and macro-F1 of **0.8282 ± 0.0316**, outperforming EfficientNetB0 in all five validation folds. An exploratory reader study using the 100 microscopy images from the held-out BACH test set showed moderate interobserver agreement between two pathologists (Cohen’s κ = 0.5105; n = 95), whereas model-to-reader concordance was lower. The ensemble did not increase reader concordance relative to EfficientNetB0. HistoBreastCAD therefore demonstrates the feasibility of integrating established deep learning components into a source-separated WSI-oriented digital pathology workflow. However, external multi-institutional validation, adjudicated reader studies, and formal explainability assessment are required before claims of clinical generalizability or utility can be supported.

## 1 Introduction

Breast cancer remains one of the most important causes of cancer-related morbidity and mortality among women worldwide. Its global burden continues to increase, creating major clinical and operational challenges for healthcare systems, particularly in settings where timely diagnosis and specialist availability are limited (Arnold et al., 2022). Early and accurate diagnosis is essential for improving patient outcomes, guiding therapeutic decisions, and reducing the downstream burden associated with delayed treatment (Begum et al., 2024). In current clinical practice, histopathological examination of breast tissue remains the diagnostic gold standard because it enables the differentiation of normal tissue, benign lesions, carcinoma *in situ*, and invasive carcinoma (He et al., 2020).

Despite its central role in breast cancer diagnosis, histopathological assessment remains highly dependent on expert interpretation. The review of hematoxylin and eosin-stained slides requires considerable training and experience, and it is affected by workload, fatigue, and interobserver variability (Rashmi et al., 2022; Guo et al., 2022). As pathology laboratories increasingly adopt digital workflows, conventional glass slides are being converted into whole-slide images (WSIs), enabling remote consultation, computational analysis, and large-scale digital archiving (Litjens et al., 2017). However, WSIs are gigapixel-scale images, which makes their manual inspection time-consuming and their automated analysis computationally demanding (Jiang et al., 2024).

Recent advances in deep learning have shown strong potential to support medical image analysis, including breast histopathology (Jiang et al., 2024; Jiménez-Gaona et al., 2020). Convolutional neural networks have been successfully applied to patch-level classification, nuclei analysis, lesion detection, and multiclass tissue categorization tasks (Bahadir et al., 2024; Balasubramanian et al., 2024; Aresta et al., 2019; Talo, 2019; Graham et al., 2019; Spanhol et al., 2016). Public datasets such as BACH have helped standardize experimentation in this field by providing both classification images and whole-slide annotations for segmentation-related tasks (Aresta et al., 2019). In parallel, transfer learning has become especially valuable in digital pathology because it improves performance in limited-data settings and allows robust feature extraction from histological images (Talo, 2019; Hameed et al., 2020; Srikantamurthy et al., 2023).

A second important research trend has been the use of segmentation methods to identify diagnostically relevant regions before classification. Architectures such as U-Net, DeepLabV3+, and related semantic segmentation models have shown promising performance in histopathological image analysis by isolating tissue regions of interest and reducing background interference (Chen et al., 2018; Priego-Torres et al., 2020; Drioua et al., 2023; Graham et al., 2019; Chen et al., 2022). Likewise, ensemble classification strategies have been proposed to improve robustness and generalization by combining predictions from independently trained convolutional architectures (Balasubramanian et al., 2024; Drioua et al., 2023; Hameed et al., 2020; Hameed et al., 2022). Previous breast histopathology studies have demonstrated that ROI localization, segmentation, and diagnostic classification can be combined within integrated WSI analysis pipelines (Gecer et al., 2018; Wu et al., 2020; Hsu et al., 2024). These studies established the feasibility of sequential localization–classification strategies and spatially resolved diagnostic analysis. At the same time, other investigations have evaluated segmentation and classification as distinct experimental tasks, even when both are addressed within the same breast histopathology study (Aresta et al., 2019; Kohl et al., 2018). Therefore, the present study does not claim that combining segmentation and classification constitutes a novel algorithmic paradigm. Instead, HistoBreastCAD focuses on the implementation and evaluation of these established components as interconnected stages of a WSI-oriented CAD workflow that includes tissue-region selection, four-class histopathological categorization, spatial reconstruction of predictions, and interactive visualization.

Recent breast histopathology research has continued to explore task-specific CNN and hybrid classification strategies. Pradeepa et al. combined EfficientNetV2 feature extraction with GRU-based sequential modeling and an attention mechanism for breast histopathology classification (Pradeepa et al., 2025). Yusuf et al. proposed an enhanced shallow CNN aimed at reducing computational complexity while performing multiclass classification across the different magnifications of the BreakHis dataset (Yusuf et al., 2025). HistoDX used an EfficientNetV2-B3-based architecture for invasive ductal carcinoma detection, incorporating normalization, augmentation, and class-balancing strategies (Arshad et al., 2025). On the BACH dataset, Ogundokun et al. combined ResNet50-based deep feature extraction with multiple machine-learning classifiers and an optimized soft-voting ensemble (Ogundokun et al., 2025). These approaches illustrate that recent breast histopathology research continues to investigate CNN-based feature extraction, hybrid modeling, and ensemble strategies, although their primary endpoint is generally image-level classification rather than an integrated segmentation–classification WSI workflow.

In parallel, transformer-based and pathology foundation-model approaches have expanded the methodological landscape. Yılmaz et al. systematically compared nearly 30 CNN and Vision Transformer architectures using the BACH and BUSI datasets, demonstrating the growing use of transformer-based representations for breast-image classification (Yilmaz et al., 2025). At a broader computational-pathology level, UNI introduced large-scale self-supervised pretraining on more than 100 million histopathology images from over 100,000 WSIs (Chen et al., 2024), whereas Prov-GigaPath extended this paradigm to whole-slide modeling through a vision-transformer-based architecture pretrained on more than 1.3 billion tiles from 171,189 slides (Xu et al., 2024). These foundation-model approaches differ substantially from HistoBreastCAD: rather than learning task-specific CNN representations exclusively from the target dataset, they leverage large-scale pretraining to generate transferable pathology representations and, in the case of Prov-GigaPath, explicitly model contextual information across WSI. HistoBreastCAD does not evaluate these architectures; its contribution is instead focused on the integration of established task-specific CNN components into a segmentation–classification–visualization workflow.

From a healthcare analytics perspective, the relevance of an AI-based pathology system extends beyond the predictive performance of individual models. Its practical value also depends on how computational outputs can support slide analysis and expert decision-making within the pathology workflow. Previous CAD and computational pathology studies have emphasized the role of AI as a decision-support approach for organizing computational image analysis and assisting specialist review (Guo et al., 2022; Campanella et al., 2019). In this context, HistoBreastCAD was designed as a workflow-oriented CAD framework in which tissue-region segmentation, multiclass classification, spatial visualization of predictions, and interactive WSI review are implemented as connected components of the same analytical system.

Integrated breast histopathology CAD pipelines have already been reported. Gecer et al. combined multiscale ROI localization with patch classification and fused the resulting maps for WSI-level interpretation (Gecer et al., 2018). Similarly, MLCD integrated ROI detection, semantic segmentation, and four-class diagnostic classification within a unified software package for breast biopsy WSIs (Wu et al., 2020). Hsu et al. further proposed a hierarchical CAD framework in which lesion detection was used for region proposal before subsequent lesion classification (Hsu et al., 2024). Therefore, HistoBreastCAD does not claim novelty in ROI-guided classification or in the individual deep-learning architectures employed. Instead, its contribution lies in the implementation and evaluation of a BACH-based workflow that connects DeepLabV3+ tissue-region segmentation, four-class classification using a frozen EfficientNetB0 + ResNet18-GAP-attention ensemble, spatial mapping of model predictions back to the WSI, and interactive web-based review within the same CAD environment.

Accordingly, the present study aims to: (i) prepare breast histopathology data suitable for leakage-safe model development and evaluation; (ii) develop and evaluate deep learning models for tissue segmentation and four-class histopathology classification; and (iii) assess model performance using quantitative cross-validation metrics together with an exploratory reader-concordance analysis. Together, these objectives are intended to evaluate the feasibility of an integrated and operationally oriented CAD workflow for digital breast pathology.

## 2 Materials and methods

HistoBreastCAD integrates DeepLabV3+ tissue-region segmentation, four-class histopathology classification, spatial mapping of model predictions, and interactive WSI visualization within a unified analytical workflow. The final classification component uses a frozen equal-weight soft-voting ensemble of EfficientNetB0 and ResNet18-GAP-attention to predict normal tissue, benign lesion, carcinoma *in situ*, or invasive carcinoma. The web-based interface displays tissue-detection information and spatially aligned classification-probability overlays on WSIs, while maintaining manual annotations as a separate user-generated layer.

### 2.1 Study design and analytical framework

This study followed a quantitative, applied, and technological design aimed at developing and evaluating an integrated computer-aided diagnosis (CAD) framework for whole-slide breast histopathology analysis. The analytical workflow combined semantic segmentation, four-class classification, spatial visualization of model predictions, and web-based WSI interaction within a unified digital pathology environment.

The methodological process was organized into sequential stages comprising dataset selection, preprocessing and quality control, source-aware patch construction, model development, quantitative evaluation under leakage-safe cross-validation, exploratory reader-concordance analysis, and integration of the analytical components within the graphical interface. Segmentation and classification were quantitatively evaluated as distinct modules using their corresponding validation protocols; therefore, their reported performance metrics should not be interpreted as a single end-to-end WSI diagnostic-performance estimate.

The overall analytical workflow of HistoBreastCAD is summarized in Figure 1. The framework was designed to connect model development and quantitative evaluation with spatial presentation of computational outputs and interactive WSI review. This organization is consistent with the decision-support orientation of CAD and healthcare analytics systems (Guo et al., 2022; Jiménez-Gaona et al., 2020; Labrada and Barkana, 2023).

**FIGURE 1:**
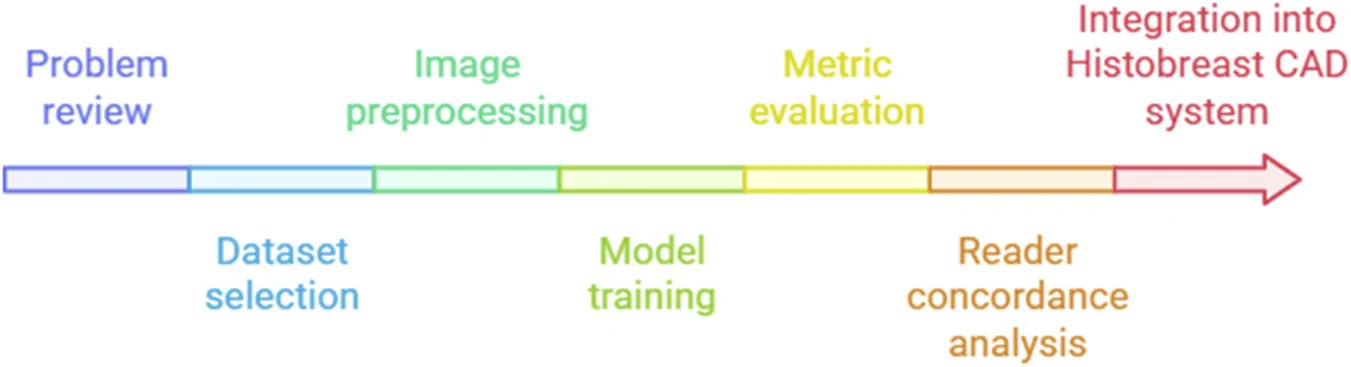
General methodological workflow of the HistoBreastCAD study.

### 2.2 Dataset and source-level partitioning

This study used the publicly available BACH dataset, originally released for the ICIAR 2018 Grand Challenge on Breast Cancer Histology Images (Aresta et al., 2019). The dataset includes complementary material for segmentation and classification, including whole-slide images (WSIs) with XML region annotations and 400 labeled breast histology images for multiclass classification. Ten annotated WSIs were used in the segmentation experiments, whereas the classification subset comprised 400 images distributed equally across four diagnostic categories: normal tissue, benign lesion, carcinoma *in situ*, and invasive carcinoma, with 100 images per class (Aresta et al., 2019).

The diagnostic categories used in the classification dataset are summarized in Table 1. Representative examples of the image types and diagnostic classes are shown in Figure 2, including one WSI and examples corresponding to normal tissue, benign lesion, carcinoma *in situ*, and invasive carcinoma. These four diagnostic categories were preserved throughout the classification experiments to maintain consistency with the original BACH dataset definition and previous studies based on this benchmark (Aresta et al., 2019).

**TABLE 1:** Diagnostic categories used in the classification dataset.

Label	Type	Description	No. of images
0	Normal tissue	Histologically normal breast tissue	100
1	Benign lesion	Non-malignant breast tissue alterations	100
2	Ductal Carcinoma *in situ*	Malignant epithelial cells confined to the ducts	100
3	Invasive Carcinoma	Malignant cells infiltrating adjacent stromal tissue	100

**FIGURE 2:**
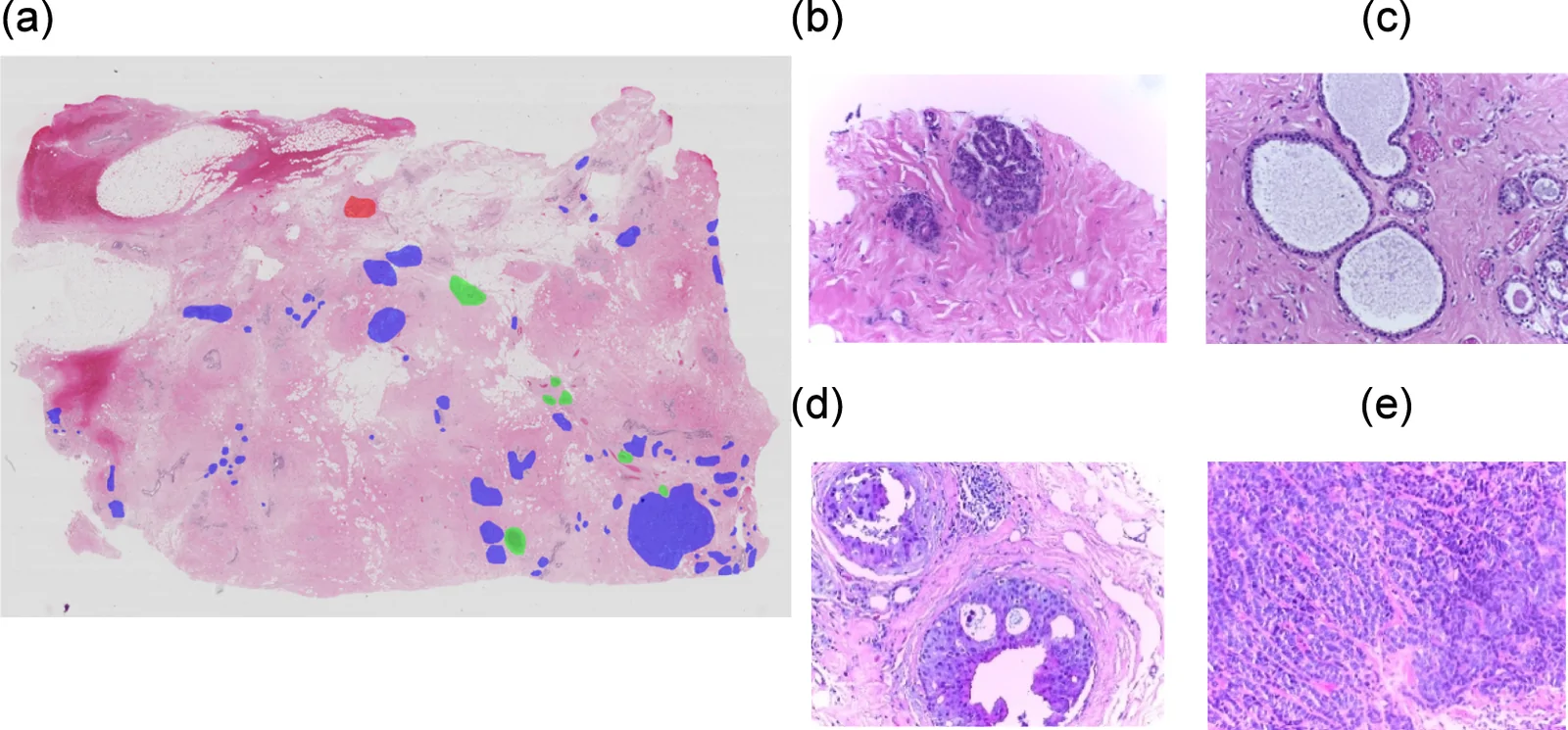
Representative examples of whole-slide images (WSIs) and corresponding image patches. **(a)** Whole-slide image (WSI); **(b)** normal tissue; **(c)** benign lesion; **(d)** *in situ* carcinoma; **(e)** invasive carcinoma.

Because multiple image patches were derived from the same source images or WSIs, data partitioning was performed at the source-image level rather than at the patch level. This was necessary to prevent spatially related patches originating from the same source image from appearing in both training and validation subsets. Separate source-level partitioning strategies were therefore applied to the segmentation and classification experiments, as described below.

For segmentation, performance was evaluated using five-fold cross-validation grouped by source WSI. The ten annotated WSIs were distributed across five folds, with two complete WSIs assigned to validation in each fold and the remaining eight used for training. Each WSI therefore appeared in the validation partition exactly once. Training and validation slide identifiers were explicitly checked in every fold, and no WSI overlap was detected.

For classification, the predefined partition of the 400 labeled source images was preserved before patch-level model development. A total of 320 source images were assigned to training and 80 to validation. Forty-eight non-overlapping 256 × 256-pixel patches were extracted from each source image, corresponding to 15,360 training patches and 3,840 validation patches. All patches derived from the same source image remained within the same partition, resulting in zero source-image overlapping between training and validation. The validation subset contained 960 patches from each of the four diagnostic categories.

### 2.3 Data preprocessing and quality control

All WSI annotations were parsed from the corresponding XML files to extract polygon coordinates, lesion labels, and associated region metadata. The annotation labels were converted into numerical indices during preprocessing and subsequently binarized for the segmentation experiments into target tissue and non-target/background regions. Non-annotated regions were assigned to the background class. Annotation geometry and label consistency were checked during dataset construction to preserve correspondence between each extracted tile, its segmentation mask, and its source WSI.

Quality-control procedures were applied during patch construction to reduce the contribution of non-informative background regions. Tissue content was estimated using thresholding in the HSV color space, allowing tiles to be dominated by white background or containing insufficient tissue to be excluded during dataset preparation. The resulting LMDB metadata were additionally audited to verify tile readability, expected label presence, source-slide identifiers, and class distributions before model training. The resulting segmentation tile inventory at both pyramid levels is summarized in Table 2.

**TABLE 2:** Segmentation tile inventory after preprocessing.

Pyramid level	Total tiles	Positive tiles*	Negative tiles*
L0	248,501	86,020	162,481
L1	16,421	5,462	10,959

* Positive and negative refer to the tile-level mask-presence categories used for balanced training sampling; segmentation performance was evaluated at the pixel level.

These preprocessing and quality-control procedures produced traceable tile collections at both pyramid levels while reducing the contribution of non-informative background. Importantly, tile metadata retained the source WSI identifier required for the slide-grouped cross-validation procedure described above. Training-specific balancing and augmentation were applied only after source-level partitioning, as detailed in Section 2.5.

### 2.4 Patch-based dataset construction

To enable computationally efficient analysis of high-resolution WSIs, segmentation data were converted into fixed-size image tiles at two pyramid levels. Level 0 (L0) corresponded to the native WSI resolution, whereas Level 1 (L1) represented the next downsampled pyramid level. At both levels, non-overlapping 256 × 256-pixel tiles were extracted using a stride of 256 pixels. This procedure produced 248,501 tiles at L0 and 16,421 tiles at L1 from the same set of ten annotated WSIs. Each extracted tile retained its source-slide identifier and spatial coordinates to preserve traceability to the original WSI. The overall patch-extraction procedure is illustrated schematically in Figure 3.

**FIGURE 3:**
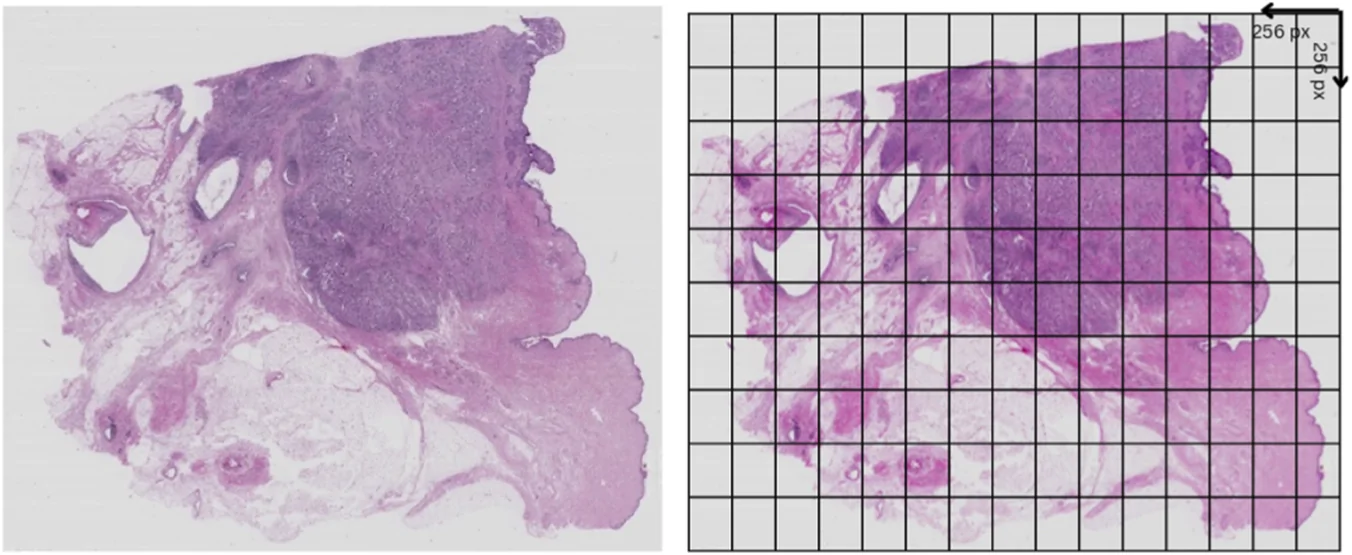
Schematic representation of the patch-extraction process from whole-slide images (WSIs). Segmentation patches were extracted independently at pyramid Levels 0 and 1 using 256 × 256-pixel tiles. The illustration is schematic and not drawn to scale.

For each segmentation tile, the corresponding annotation mask was generated from the WSI XML regions and stored together with the image tile. Tiles were subsequently categorized as positive or negative according to the presence of annotated target pixels in the corresponding mask for the purpose of training-time class balancing. Tissue content was estimated using HSV-based thresholding to reduce the contribution of tiles dominated by blank background. Importantly, the segmentation task itself remained pixel based; the positive/negative tile designation was used only for sampling and did not replace the pixel-level reference masks used for training and evaluation.

Segmentation tiles, binary masks, and associated metadata were stored in Lightning Memory-Mapped Database (LMDB) format to support efficient random access during training and validation. Metadata included tile coordinates, pyramid level, patch size, tissue information, and the corresponding slide_id. The source-slide identifier was retained as a mandatory metadata field because it was subsequently used to construct and audit the WSI-grouped cross-validation folds.

For the classification stage, each of the 400 labeled histopathology source images was divided into 48 non-overlapping 256 × 256-pixel patches. Importantly, source images were assigned to the predefined training or validation partition before patch-level model development, and all patches originating from a given source image remained within the same partition. This procedure generated 19,200 CSV-indexed patches in total: 15,360 patches derived from 320 training source images and 3,840 patches derived from 80 held-out validation source images. Classification metadata were stored in a CSV file containing the patch path, diagnostic label, source-image identifier, predefined split, and spatial coordinates, as illustrated in Table 3.

**TABLE 3:** Structure of the classification patch index.

Split	Label	patch_relpath	source_image	Col	Row
train	*InSitu*	train/*InSitu*/is079_c00_r00.png	is079.tif	0	0
train	Invasive	train/Invasive/iv066_c03_r02.png	iv066.tif	3	2
train	Normal	train/Normal/n007_c06_r00.png	n007.tif	6	0
train	Benign	train/Benign/b040_c07_r00.png	b040.tif	7	0

In addition to the CSV-derived training patches, WSI-derived LMDB, tiles were incorporated exclusively into the classification training pool. Their filtering and class-balancing procedure is described in Section 2.5; no LMDB-derived tile was included in the classification validation set.

### 2.5 Data augmentation and class balancing

Data augmentation was applied only after source-level partitioning and exclusively to training samples. Validation samples were never augmented. For the segmentation experiments, training-time augmentation consisted of random horizontal and vertical flips applied jointly to the image tile and its corresponding segmentation mask. No augmentation was applied during validation. For classification, augmentation was generated on-the-fly from training samples only, whereas the held-out validation images were processed without stochastic augmentation. Classification inputs were normalized using the ImageNet mean and standard deviation values to maintain compatibility with the pretrained convolutional backbones (Talo, 2019). This ordering ensured that augmented versions of a sample could not appear across training and validation partitions.

For segmentation, class balancing was performed independently within the training portion of each cross-validation fold. Tiles were categorized as positive or negative according to the presence of target pixels in their reference masks, and each training epoch was constructed using equal number of positive and negative tiles sampled without replacement. At L0, each fold used a fixed training budget of 50,000 tiles per epoch, comprising 25,000 positive and 25,000 negative tiles. At L1, the same 1:1 sampling principle was applied, but the per-epoch budget was capped by the number of available positive tiles in each training fold. Validation data were neither balanced nor subsampled; all tiles belonging to the validation WSIs were evaluated. Hard-negative oversampling was disabled in the final cross-validation experiments. The segmentation sampling strategy at both pyramid levels is summarized in Table 4.

**TABLE 4:** Segmentation training sampling and validation protocol.

Level	Total tiles	Positive tiles	Negative tiles	Training sampling per epoch	Validation
**L0**	248,501	86,020	162,481	50,000 (25,000 positive +25,000 negative)	All tiles from held-out WSIs
**L1**	16,421	5,462	10,959	Balanced 1:1; fold-specific budget of 6,554–10,170 tiles	All tiles from held-out WSIs

For classification, balancing was also restricted to the training partition. The predefined CSV training subset contained 15,360 patches derived from 320 source images, while 3,840 patches from 80 held-out source images were reserved for validation. WSI-derived L0 tiles were subsequently filtered and equalized by class before being added exclusively to the training pool. This procedure contributed 10,888 additional LMDB tiles, resulting in a development training set of 26,248 samples, with 6,562 samples per diagnostic class. The validation set remained unchanged at 3,840 patches, with 960 samples per class, and contained no LMDB-derived tiles. The resulting training and validation distributions are summarized in Table 5.

**TABLE 5:** Classification sample distribution in the source-level development split.

Class	Label	Training	Validation	Total
Normal	0	6,562	960	7,522
Benign	1	6,562	960	7,522
*In situ*	2	6,562	960	7,522
Invasive	3	6,562	960	7,522
Total	-	26,248	3,840	30,088

Accordingly, all balancing and stochastic augmentation procedures were confined to the training data and were performed only after the source-level partitions had been established. Neither validation WSIs in the segmentation experiments nor held-out classification source images were subjected to training-time sampling or augmentation. This separation was maintained throughout model development to prevent information leakage between training and validation data.

### 2.6 Segmentation model development

The segmentation module was designed to identify tissue regions of interest for subsequent analysis within the HistoBreastCAD workflow. The final standardized segmentation experiments compared three semantic segmentation architectures: U-Net with a ResNet-34 encoder, DeepLabV3 with a ResNet-50 backbone, and DeepLabV3+ with a ResNet-50 backbone. All three architectures were evaluated separately at pyramid Levels 0 (L0) and 1 (L1) using leakage-safe, WSI-grouped five-fold cross-validation. Fold assignments were held constant across architectures within each pyramid level, although the L0 and L1 fold compositions were not identical. DeepLabV3+ achieved the highest out of fold (OOF) micro-Dice among the evaluated architectures at both pyramid levels and was retained as the segmentation architecture used in the final workflow. Preliminary experiments also considered Mask R-CNN; however, because this configuration was not re-evaluated under the final standardized cross-validation protocol, it was excluded from the quantitative architecture comparison.

DeepLabV3+ was implemented with a ResNet-50 encoder and trained for binary semantic segmentation, with the target defined as annotated tissue of interest versus non-target/background pixels. This formulation reflects the role of the segmentation component within HistoBreastCAD: segmentation is used to spatially identify tissue regions for subsequent analysis rather than to assign the final four-class diagnostic label. The DeepLabV3+ architecture combines atrous convolutions with atrous spatial pyramid pooling (ASPP) to capture contextual information at multiple receptive-field scales, while its decoder progressively recovers spatial information for segmentation output (Chen et al., 2018; Priego-Torres et al., 2020).

Model training used a combined Dice and focal loss, with equal weighting of both components. Optimization was performed using AdamW with an initial learning rate of 3 × 10^−4^ and weight decay of 1 × 10^−4^. A maximum of 40 epochs was allowed, with learning-rate reduction and early stopping based on validation performance. Mixed-precision training was used throughout. Batch size was 16 for U-Net and DeepLabV3 and 32 for DeepLabV3+; early stopping used a patience of eight epochs. In the final DeepLabV3+ L0 experiment, checkpoint selection, learning-rate scheduling, and early stopping were governed by pixel-level micro-averaged Dice computed over the complete validation partition of each fold at a fixed threshold of 0.70.

Micro-averaged Dice was adopted as the checkpoint-selection criterion because averaging Dice independently across tiles can be strongly influenced by empty-background tiles. In particular, a tile containing neither reference-positive nor predicted-positive pixels may receive a perfect per-tile Dice score despite providing no information about lesion segmentation. To avoid this effect, validation metrics in the final evaluation were derived from pooled pixel-level confusion counts across the complete validation partition. The previous tile-averaged Dice values were therefore not used as the primary segmentation performance estimates in the revised analysis. For the standardized architecture comparison, the retained checkpoints had originally been selected using the tile-averaged validation Dice and were subsequently re-evaluated using pooled pixel-level metrics. Therefore, these runs provide a common descriptive comparison of the three architectures under the same evaluation convention. The final DeepLabV3+ L0 configuration was subsequently re-trained with micro-Dice used directly for checkpoint selection, learning-rate scheduling, and early stopping.

Segmentation performance was quantified using Precision, Recall, Intersection over Union (IoU), and Dice coefficient. All four metrics were calculated from the same pooled pixel-level counts of true positives (TP), false positives (FP), and false negatives (FN):

$$Precision=\frac{TP}{TP+FP}$$

$$Recall=\frac{TP}{TP+FN}$$

$$IoU=\frac{TP}{TP+FP+FN}$$

$$Dice=\frac{2TP}{2TP+FP+FN}$$

Consequently, the reported Dice and IoU values satisfy the mathematical identity $IoU=Dice/\left(2-Dice\right)$ . A fixed binarization threshold of 0.70 was used throughout the segmentation evaluation and was not optimized on the validation folds.

Two complementary cross-validation summaries were reported. First, out-of-fold (OOF) micro-averaged metrics were obtained by pooling TP, FP, and FN across all five validation folds, so that each of the ten WSIs contributed exactly once to the final evaluation. Second, fold-level micro-Dice values were summarized as mean ± standard deviation to characterize between-partition variability. The OOF value and the unweighted fold mean are not expected to be numerically identical because the former is weighted implicitly by the number of evaluated pixels in each fold.

For WSI-level inference, the complete gigapixel image was not loaded into GPU memory. Instead, the slide was accessed using OpenSlide and processed patch-wise at pyramid Level 0 using 256 × 256-pixel tiles with a stride of 128 pixels, corresponding to 50% overlap between adjacent tiles. Tiles were processed in mini-batches, making GPU memory requirements dependent on the batch and tile dimensions rather than on the full WSI dimensions. DeepLabV3+ generated a pixel-level probability map for each tile. For the visualization presented in Figure 4e, each probability map was summarized using its 95th percentile (p95), yielding one segmentation score per spatial tile location. The resulting score grid was resized to the WSI thumbnail and thresholded at 0.70 to produce the displayed segmentation-probability overlay. Full-resolution pixel-wise stitching was therefore not required for this visualization.

**FIGURE 4:**
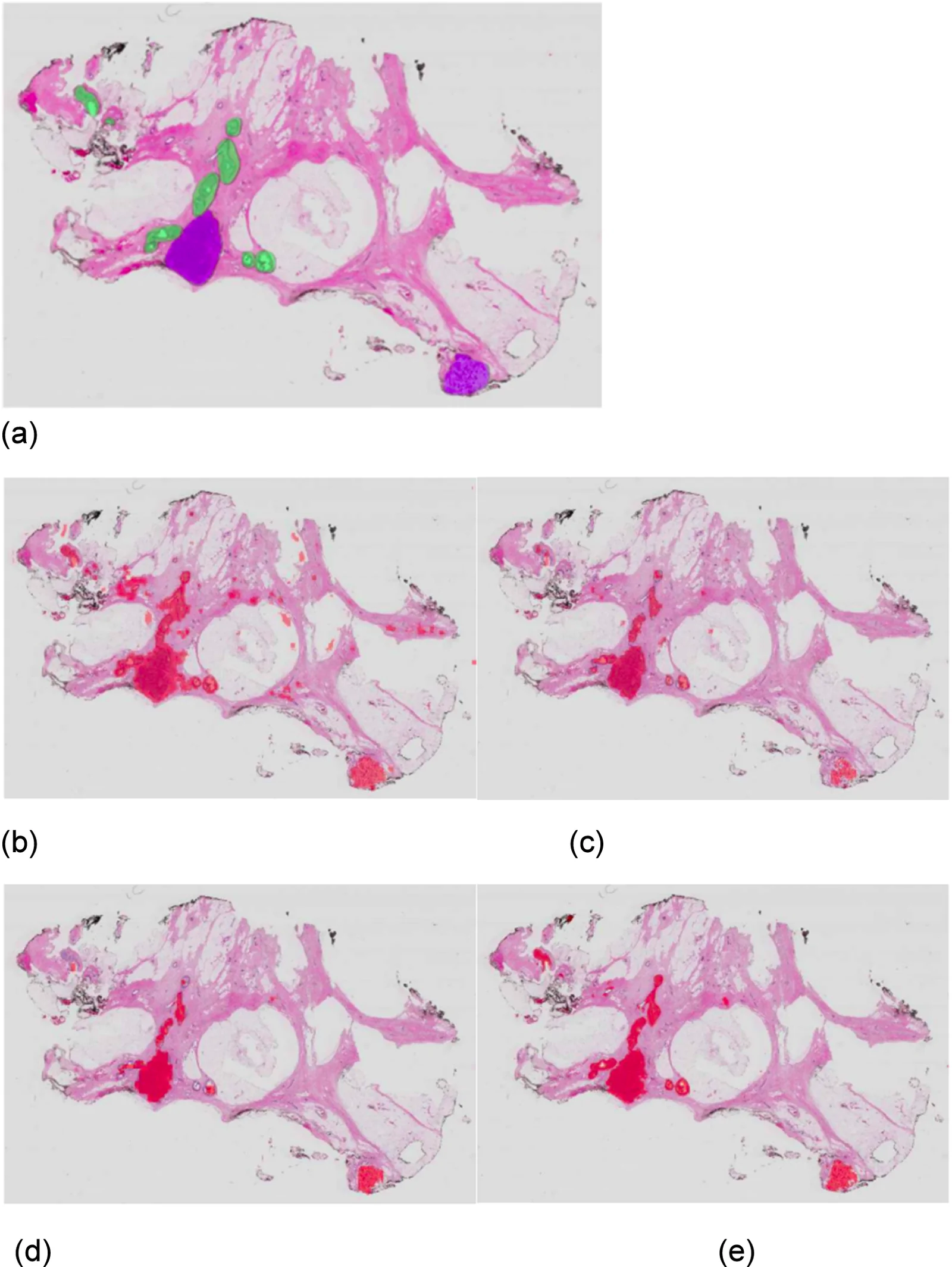
Representative WSI-level segmentation outputs and spatial visualization. **(a)** Ground-truth annotation; **(b)** U-Net at L1; **(c)** U-Net at L0; **(d)** DeepLabV3 at L1; and **(e)** DeepLabV3+ at L0 displayed as a tile-level segmentation-probability overlay.

Initial system development and WSI inference were performed on a workstation equipped with an NVIDIA RTX 2080 Ti-class GPU with 11 GB of VRAM, an Intel Core i9-10980HK processor, and 32 GB of RAM. The final leakage-safe L0 cross-validation and micro-Dice checkpoint-selection experiments were independently executed on an NVIDIA GeForce RTX 4080 with 16 GB of VRAM, an Intel Core i7-13700F processor, and 31 GB of RAM using PyTorch 2.5.1 and CUDA 12.1-compatible binaries. Because WSI inference was patch based, neither configuration required loading an entire WSI into GPU memory. The micro-averaged re-evaluation of the three semantic segmentation architectures is summarized in Table 6.

**TABLE 6:** Leakage-safe five-fold cross-validation performance of the evaluated segmentation architectures.

Model	Level	OOF precision	OOF recall	OOF IoU	OOF micro-dice	Fold micro-dice mean ± SD
**DeepLabV3+**	L1	0.9094	0.8058	0.7459	**0.8545**	0.7653 ± 0.1721
**DeepLabV3**	L1	0.9169	0.7848	0.7326	0.8457	0.7668 ± 0.1579
**U-Net**	L1	0.9225	0.6436	0.6106	0.7582	0.6038 ± 0.2812
**DeepLabV3+**	L0	0.9039	0.7828	0.7227	**0.8390**	0.7812 ± 0.1415
**DeepLabV3**	L0	0.9085	0.7587	0.7049	0.8269	0.7640 ± 0.1271
**U-Net**	L0	0.9301	0.6571	0.6262	0.7701	0.6088 ± 0.3204

OOF, metrics were recomputed from pooled pixel-level confusion counts. Checkpoints in this standardized architecture comparison were originally selected using tile-averaged validation Dice and subsequently re-evaluated using micro-averaged metrics. L0 and L1 used different fold compositions; therefore, the values across pyramid levels should not be interpreted as a paired level-to-level comparison.

After the standardized architecture comparison, DeepLabV3+ at L0 was re-trained using micro-Dice directly as the checkpoint-selection criterion. This final run obtained an OOF micro-Dice of 0.8390, IoU of 0.7226, Precision of 0.8278, and Recall of 0.8505, with a fold-level micro-Dice of 0.7967 ± 0.1097. The unchanged OOF Dice relative to the original DeepLabV3+ L0 run indicates that changing the selection criterion did not increase the aggregate score, but shifted the operating point toward higher sensitivity and removed the empty-tile failure mode associated with tile-averaged Dice. Although the L1 DeepLabV3+ experiment yielded a nominally higher OOF micro-Dice, L0 and L1 used different cross-validation fold compositions and were therefore not treated as a paired resolution comparison. The L0 configuration was retained for the final WSI workflow to preserve native-resolution spatial detail during patch-wise inference and visualization.

Representative WSI-level segmentation outputs are shown in Figure 4, while the fold-level variability of the evaluated semantic segmentation architectures is presented in Figure 5. DeepLabV3+ achieved the highest OOF micro-Dice among the three architectures at both evaluated pyramid levels, although substantial between-fold variability was observed.

**FIGURE 5:**
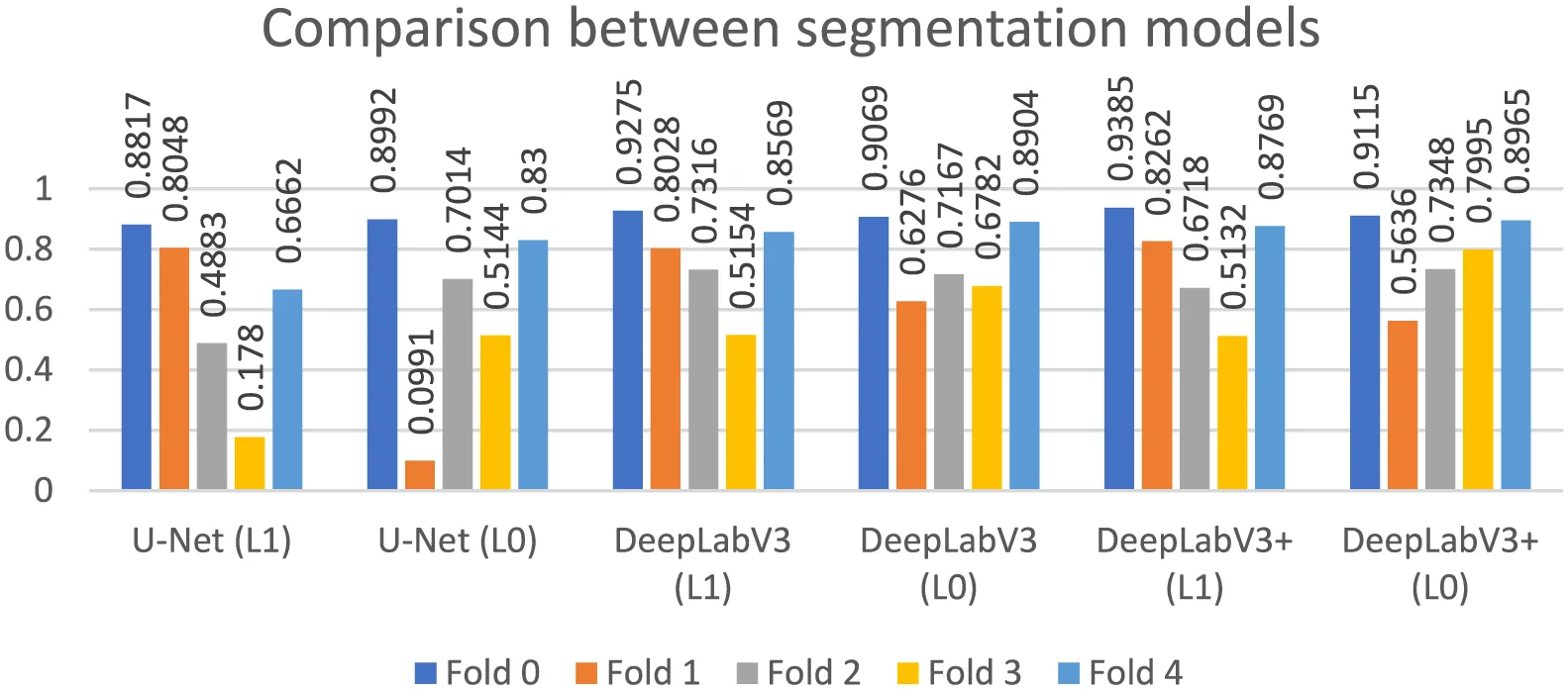
Fold-level variability in micro-Dice across the evaluated semantic segmentation architectures at pyramid Levels L0 and L1. Each value represents one held-out WSI-grouped validation fold. L0 and L1 used different fold compositions and are therefore shown as separate cross-validation analyses rather than as a paired resolution comparison.

### 2.7 Classification model development

The classification module was designed to assign histopathological image patches to one of four diagnostic categories: normal tissue, benign lesion, carcinoma *in situ*, and invasive carcinoma. Within the complete HistoBreastCAD workflow, this classifier is subsequently applied to tissue regions selected during WSI analysis. Transfer learning with ImageNet-pretrained convolutional backbones was adopted because of the limited amount of labeled histopathological material and the demonstrated utility of pretrained representations in medical image classification (Jiménez-Gaona et al., 2020; Talo, 2019).

Four classification architectures were evaluated under the same leakage-controlled data protocol: EfficientNetB0, ResNet18, ResNet18-GAP-attention, and DarkNet19-GAP-attention. EfficientNetB0 uses compound scaling of network depth, width, and input resolution (Tan and Le, 2019), whereas ResNet18 is based on residual connections that facilitate optimization of deep convolutional networks (He et al., 2016). The GAP-attention variants combine global average pooled features with an attention-derived representation before the final classification head, allowing both global and spatially weighted feature information to contribute to the prediction.

All four classifiers were trained using 256 × 256-pixel inputs and focal loss. The audited development experiments used a batch size of 64 and a maximum of 25 epochs. Model checkpoints were selected according to the highest macro-averaged F1-score observed on the held-out validation partition rather than according to the final training epoch. This distinction was important because several architectures reached their best validation performance before the final epoch. All performance values reported for model selection were therefore obtained by reloading and re-evaluating the corresponding best checkpoint. The principal training parameters used in the audited classification experiments are summarized in Table 7.

**TABLE 7:** Training configuration used for the classification experiments.

Parameter	Value
Input size	256 × 256 pixels
Diagnostic classes	4
Batch size	64
Maximum epochs	25
Loss function	Focal loss
Optimizer	AdamW
Model-selection metric	Macro-averaged F1-score
Data augmentation	On-the-fly, training only
Training seed	42

Initial model and ensemble selection was performed using the fixed source-image-level development split described in Sections 2.2–2.5. The four architectures were trained on identical development samples, allowing direct comparison under the same data conditions. Classification stability was subsequently assessed using source-image-grouped five-fold cross-validation, with all patches originating from the same source image retained within the same fold. The cross-validation results are reported separately to quantify between-partition variability rather than to redefine the ensemble using the reader-study material.

Equal-weight pairwise soft-voting combinations were evaluated on the fixed development validation set to examine whether models with different feature representations provided complementary predictive information. Among the evaluated combinations, the ensemble comprising EfficientNetB0 and ResNet18-GAP-attention achieved the highest development macro-F1 and was therefore selected as the classification ensemble used for subsequent system integration and pathologist-concordance analysis. The ensemble configuration was fixed before the reader annotations were used for model comparison; no alternative ensemble was selected based on reader concordance.

For each input patch x , EfficientNetB0 and ResNet18-GAP-attention independently generated class logits, which were converted into four-class probability distributions using the softmax function.

$$p_{c}^{\left(m\right)}\left(x\right)=\frac{\exp\left(z_{c}^{\left(m\right)}\left(x\right)\right)}{\sum_{j=1}^{4}\exp\left(z_{j}^{\left(m\right)}\left(x\right)\right)}$$

For each diagnostic class c , the ensemble probability was calculated as the arithmetic mean of the two model probabilities using equal weights of 0.5.

$$p_{c}^{ens}\left(x\right)=\frac{1}{2}\left[p_{c}^{EffNet}\left(x\right)+p_{c}^{ResNet-GAP-attn}\left(x\right)\right]$$

The final predicted category corresponded to the class with the highest averaged probability.

$$\hat{y}\left(x\right)=\arg\max_{c}p_{c}^{ens}\left(x\right)$$

Thus, the ensemble represents equal-weight soft voting and does not involve hard voting, feature-level fusion between the two networks, or an additional learned meta-classifier.

Equal weighting was selected as a simple, parameter-free fusion strategy that allowed both independently trained architectures to contribute to the final probability distribution without introducing additional trainable parameters or estimating fusion weights from the limited development data. Weighted probability fusion and learned stacking were not systematically evaluated in the present study; therefore, no superiority over these alternative fusion strategies is claimed.

Classification performance was assessed using accuracy and macro-averaged F1-score, with class-specific F1-scores used to examine diagnostic-category behavior. Five-fold cross-validation grouped by source image was used to quantify variability across data partitions. EfficientNetB0 achieved the highest macro-F1 among the individual architectures (0.8142 ± 0.0388), while ResNet18-GAP-attention and ResNet18 showed similar performance (0.7896 ± 0.0252 and 0.7836 ± 0.0330, respectively). The frozen EfficientNetB0 + ResNet18-GAP-attention ensemble achieved the highest overall macro-F1 (0.8282 ± 0.0316), outperforming EfficientNetB0 in all five folds. The final cross-validation results are summarized in Table 8, and fold-level variability is presented in Figure 6.

**TABLE 8:** Five-fold cross-validation performance of the evaluated classification architectures and the frozen ensemble.

Architecture	Accuracy	Macro F1	Normal F1	Benign F1	*In situ* F1	Invasive F1
EfficientNetB0 + ResNet18-GAP-attn	0.8283 ± 0.0320	0.8282 ± 0.0316	0.8151	0.8070	0.8146	0.8762
EfficientNetB0	0.8143 ± 0.0387	0.8142 ± 0.0388	0.8025	0.7891	0.7992	0.8660
ResNet18-GAP-attn	0.7900 ± 0.0263	0.7896 ± 0.0252	0.7831	0.7649	0.7746	0.8361
ResNet18	0.7837 ± 0.0332	0.7836 ± 0.0330	0.7735	0.7620	0.7727	0.8261
DarkNet19-GAP-attn	0.7397 ± 0.0305	0.7389 ± 0.0303	0.7313	0.7135	0.7161	0.7947

Accuracy and macro F1 are reported as mean ± standard deviation across the five folds. Class-specific F1 values represent the corresponding mean across folds. Validation was exactly balanced across the four diagnostic categories (960 patches per class in each fold), and accuracy and macro F1 consequently showed very similar numerical values, although they represent distinct performance measures.

**FIGURE 6:**
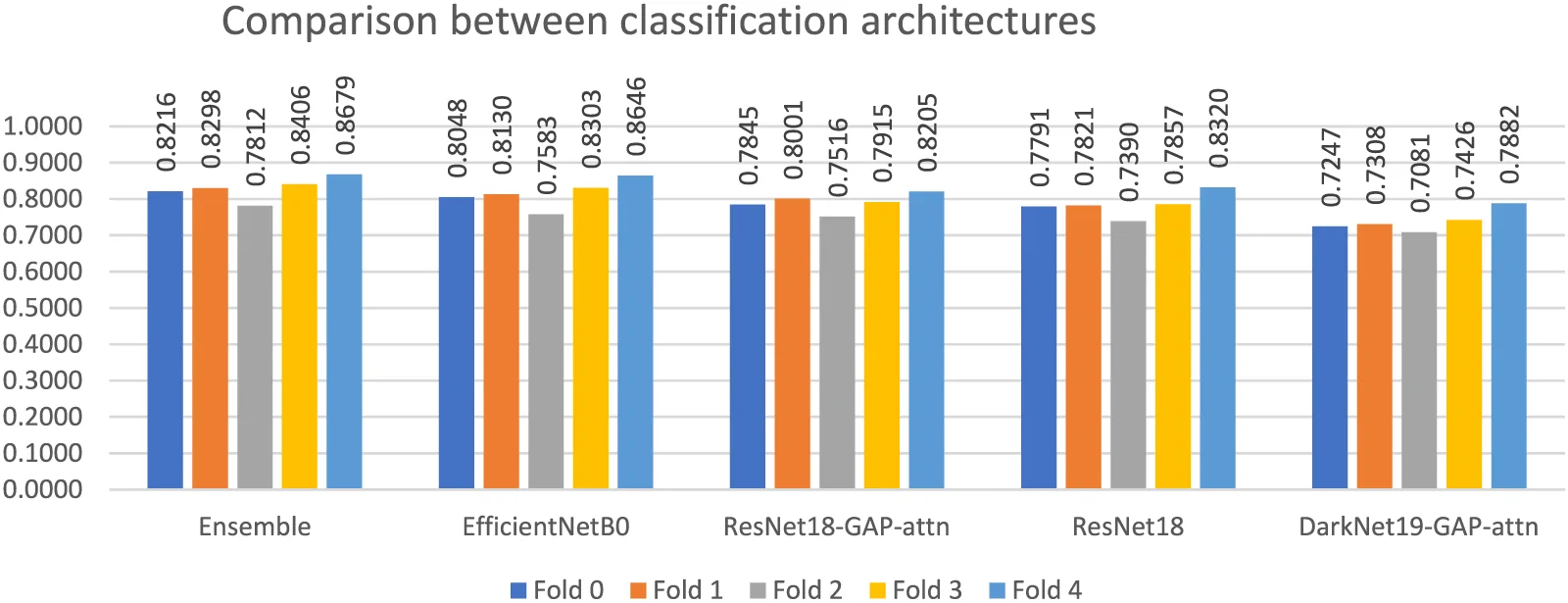
Fold-level classification performance under source-image-grouped five-fold cross-validation. Macro-averaged F1-score is shown for the four individual architectures and the frozen EfficientNetB0 + ResNet18-GAP-attention ensemble.

### 2.8 Exploratory reader-concordance analysis on the BACH test set

An exploratory reader concordance analysis was conducted to examine the agreement between classification-model predictions and independent pathologist interpretations. This analysis was designed as a complementary reader study rather than as an evaluation against a definitive clinical ground truth. The reader annotations were not used for model training, hyperparameter optimization, architecture selection, or ensemble selection. Consequently, the concordance results reported in this section should be interpreted separately from the internal classification performance estimated using the BACH development data.

The reader study comprised the 100 microscopy images included in the BACH challenge test set, each measuring 2048 × 1,536 pixels. These images are distinct from the 400 labeled BACH microscopy images used for classifier development and cross-validation.

For model inference, each test image was divided into 48 non-overlapping 256 × 256-pixel patches using a stride of 256 pixels. Patch-level class probabilities were aggregated at the image level by calculating the arithmetic mean of the probability vectors across all 48 patches, and the diagnostic category with the highest aggregated probability was assigned as the image-level model prediction. Thus, the unit compared with the pathologists was the complete microscopy image rather than an individual patch.

The BACH test images were not used during model development, architecture selection, hyperparameter optimization, or ensemble selection. Because the public test set does not provide the corresponding diagnostic reference labels, the present study did not use these images as a conventional labeled test set. Instead, they were independently interpreted by two pathologists and used exclusively for exploratory reader-concordance analysis. To additionally verify that no microscopy image from the BACH test set duplicated material used for classifier development, the 4,800 tiles derived from the 100 test images were compared at the pixel level with the 19,200 patches derived from the 400 labeled BACH development images using image hashing. No pixel-identical matches were identified.

Both pathologists were presented with the complete set of 100 images and assigned one of the four diagnostic categories used by the classification system whenever a valid interpretation could be provided. During data verification, a transcription alignment issue affecting the Reader 2 annotation record was identified and resolved before the concordance analysis. After verification, Reader 1 provided 96 valid four-class labels and Reader 2 provided 99, with 95 images having valid paired annotations from both readers.

Model predictions were available for all 100 images. Model-to-reader comparisons therefore used the valid cases available for the corresponding reader (n = 96 for Reader 1 and n = 99 for Reader 2), whereas direct interobserver agreement was calculated using only the 95 images with valid labels from both readers.

The prespecified model comparison included the selected EfficientNetB0 + ResNet18-GAP-attention ensemble and EfficientNetB0 as the best-performing individual model on the fixed development split. ResNet18-GAP-attention was additionally retained in the descriptive analysis as an ensemble component. No alternative ensemble combination, patch-aggregation rule, or model-selection strategy was optimized using the reader annotations.

Concordance was quantified primarily using Cohen’s kappa (κ), which measures categorical agreement beyond that expected by chance. Observed agreement was also reported descriptively. Because the four diagnostic categories can additionally be interpreted along an ordered progression from normal tissue to invasive carcinoma, quadratic-weighted Cohen’s kappa was calculated as a secondary concordance measure. The unweighted Cohen’s κ remained the primary metric to preserve direct assessment of agreement across the four nominal diagnostic categories.

The diagnostic distributions of the two readers and the evaluated models are summarized in Table 9.

**TABLE 9:** Distribution of pathologists and model predictions across the diagnostic categories.

Diagnostic category	Pathologist 1 (n = 96)	Pathologist 2 (n = 99)	Ensemble (n = 100)	EfficientNetB0 (n = 100)	ResNet18-GAP-attn (n = 100)
Normal tissue	10	10	35	36	33
Benign lesion	35	53	19	13	32
Carcinoma *in situ*	24	18	14	13	15
Invasive carcinoma	27	18	32	38	20
Total	96	99	100	100	100

One hundred histopathological images were presented to both readers. Reader 1 provided 96 valid four-class annotations and Reader 2 provided 99; 95 images had valid paired annotations from both readers. Model distributions correspond to predictions generated for all 100 images.

Interobserver agreement and model-to-reader concordance are further illustrated using the confusion matrices presented in Figure 7.

**FIGURE 7:**
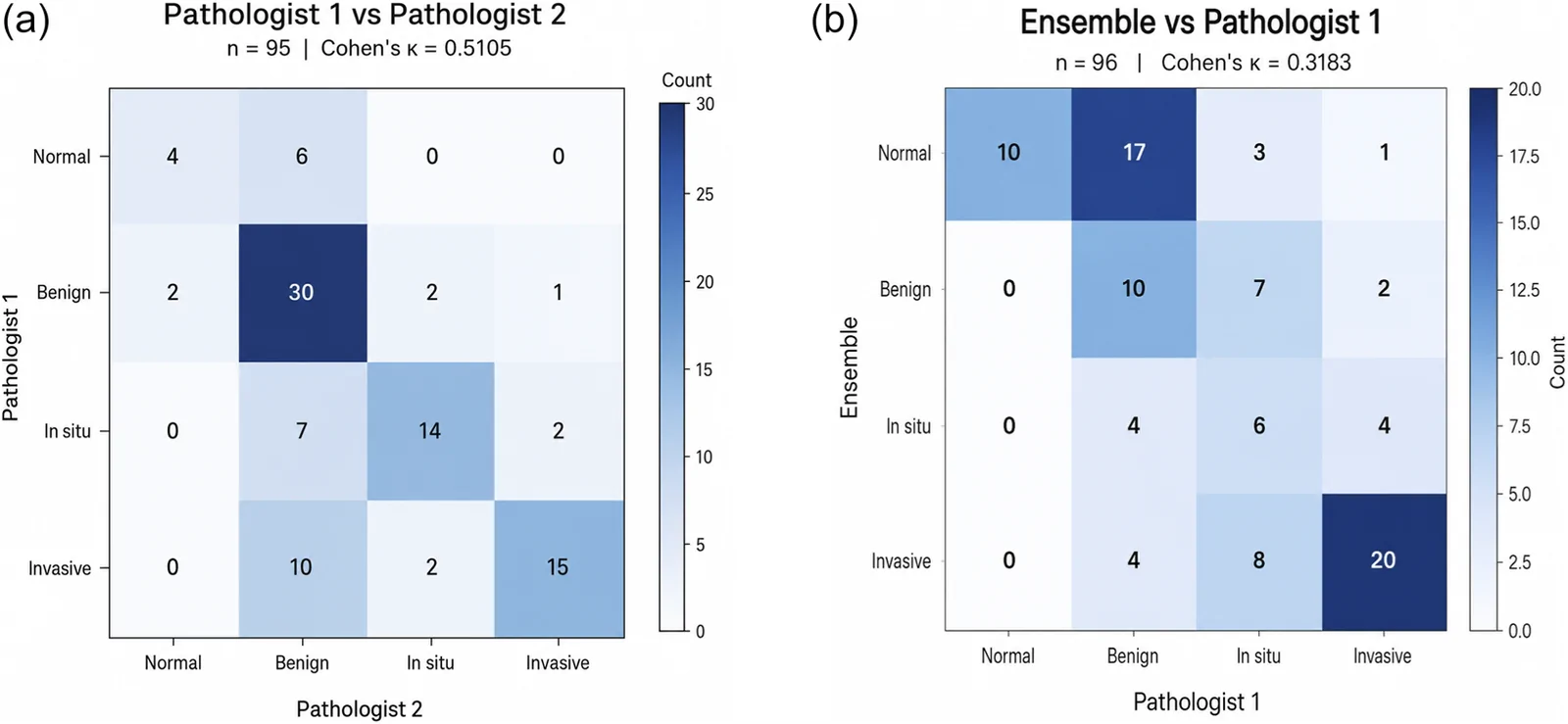
Confusion matrices from the exploratory pathologist-concordance analysis. **(a)** Comparison between Pathologist 1 and Pathologist 2 using the 95 images with valid paired annotations (Cohen’s κ = 0.5105). **(b)** Comparison between the frozen EfficientNetB0 + ResNet18-GAP-attention ensemble and Pathologist 1 using the 96 images with valid Pathologist 1 annotations (Cohen’s κ = 0.3183). Rows represent the first-listed evaluator/model and columns represent the second-listed evaluator.

This study was therefore interpreted as an exploratory concordance assessment rather than as a definitive evaluation of clinical diagnostic accuracy. No consensus diagnosis, third-reader adjudication, or independent reference standard was available to resolve reader disagreements. The reader-study material was consequently not used to estimate the internal predictive performance of the classification models. A consolidated summary of the dataset composition, source-level partitioning, balancing procedures, and reader-concordance protocol is provided in Table 10.

**TABLE 10:** Summary of dataset composition, partitioning, balancing, and reader-concordance protocol.

Component	Item	Details
Segmentation	Source material	10 annotated BACH WSIs
Segmentation	Extracted tiles	L0: 248,501 tiles (86,020 positive; 162,481 negative); L1: 16,421 tiles (5,462 positive; 10,959 negative)
Segmentation	Partitioning	Five-fold cross-validation grouped by WSI; 8 complete WSIs for training and 2 for validation per fold; each WSI used for validation once
Segmentation	Balancing	Training only, 1:1 positive/negative sampling; L0: 50,000 tiles/epoch; L1: fold-specific 6,554–10,170 tiles/epoch
Segmentation	Validation/test	All tiles from held-out WSIs evaluated; no separate test partition
Classification	Source material	400 labeled BACH microscopy images; 100 images per class
Classification	Classes	Normal, benign, carcinoma *in situ*, invasive carcinoma
Classification	Patch extraction	48 non-overlapping 256 × 256 patches/source image; 19,200 CSV-derived patches
Classification	Cross-validation	Five-fold grouping by source image; 320 training images and 80 validation images/fold
Classification	Per-fold samples	15,360 CSV training patches +10,888 LMDB training-only tiles = 26,248 training samples; 3,840 validation patches
Classification	Class distribution	Training: 6,562 samples/class; validation: 960 patches/class
Classification	Balancing	LMDB-derived training tiles filtered and equalized by class; validation unchanged and balanced
Classification	Validation/test	Every one of the 400 labeled source images appeared in validation once; no conventional labeled test set
Reader study	Source material	100 microscopy images from the BACH challenge test set, 2048 × 1,536 pixels
Reader study	Model inference	48 non-overlapping 256 × 256 patches/image; probabilities averaged across patches to produce one image-level prediction
Reader study	Readers	Two pathologists
Reader study	Annotation protocol	Each reader assigned one of the four BACH diagnostic categories whenever a valid interpretation could be provided
Reader study	Valid annotations	Reader 1: n = 96; Reader 2: n = 99; paired valid annotations: n = 95
Reader study	Agreement analysis	Observed agreement and unweighted Cohen’s κ; quadratic-weighted Cohen’s κ as a secondary measure
Reader study	Reference standard	No consensus diagnosis, third-reader adjudication, or test-set reference labels were available to the present study

### 2.9 System integration and visualization interface

Beyond model development, a web-based interface was implemented to support interactive review of model outputs in a digital pathology setting. The system followed a client-server architecture, with a React-based frontend and a FastAPI backend connected to a PyTorch-based inference engine. Whole-slide images were visualized using OpenSeadragon, which enables tile-based exploration of gigapixel pathology images through smooth pan and zoom interactions (Schüffler et al., 2021).

The interface incorporated three distinct visualization and interaction layers: a tissue-detection overlay indicating evaluable regions, a spatial classification-probability overlay derived from patch-level model predictions, and an independent manual annotation layer for expert review. For the classification overlay, each analyzed tile was assigned the color associated with the highest-probability diagnostic class, while the corresponding class probability modulated the transparency of the displayed color. The resulting color-coded overlay is spatially aligned with the WSI and is intended to help users identify and review regions associated with model predictions. It should not be interpreted as a continuous pixel-level saliency map or as a formal explainability method such as Grad-CAM or SHAP. The manual annotation layer separately allows users to delineate and label regions directly on the WSI.

The internal AI-engine architecture that connects segmentation and classification within the inference workflow is shown in Figure 8. The full client-server system architecture is presented in Figures 9, 10 illustrates the manual annotation functionality of the HistoBreastCAD interface, showing user-defined diagnostic regions drawn directly on the WSI. In practical terms, this interface layer transformed the proposed framework from a set of isolated analytical models into a usable CAD environment for digital pathology review.

**FIGURE 8:**
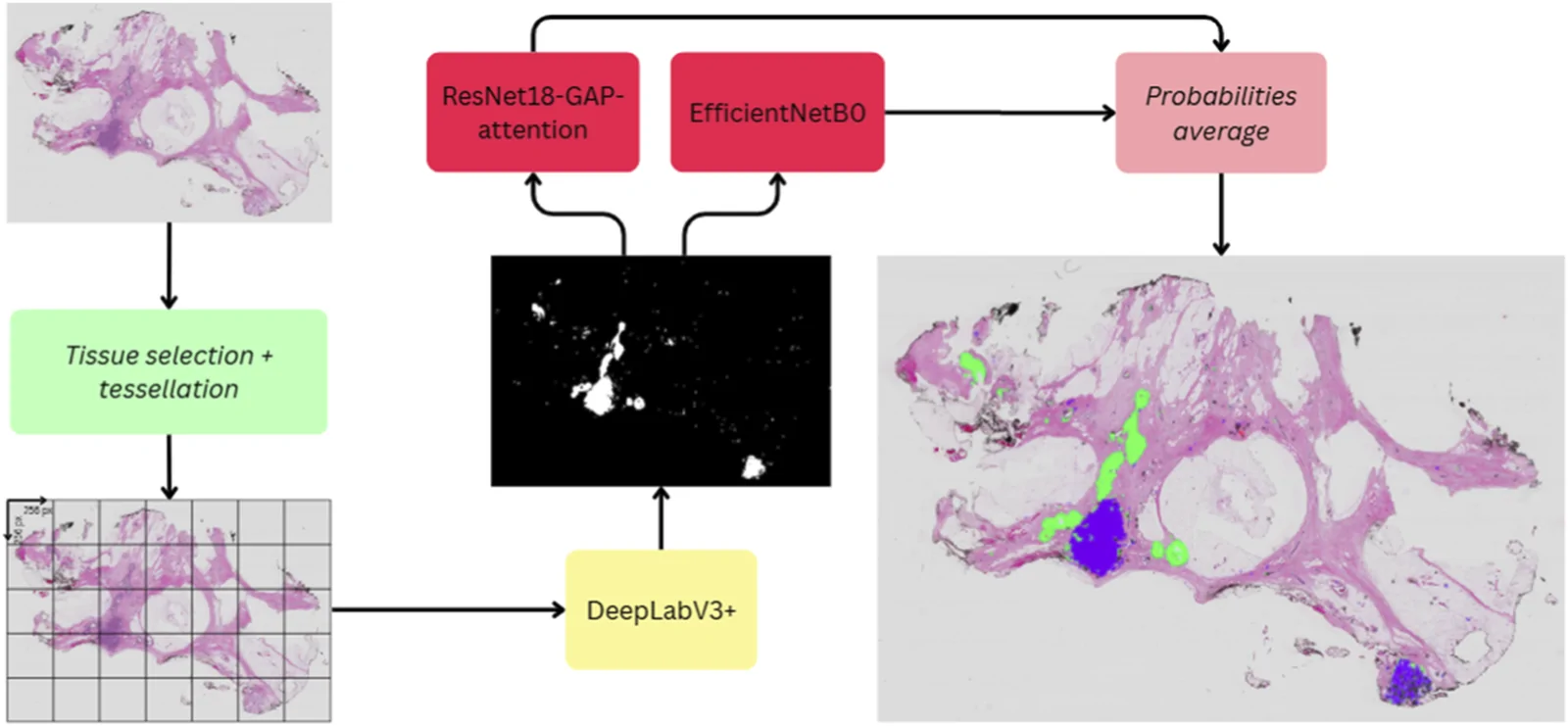
Architecture of the HistoBreastCAD AI engine integrating tissue segmentation and multiclass classification.

**FIGURE 9:**
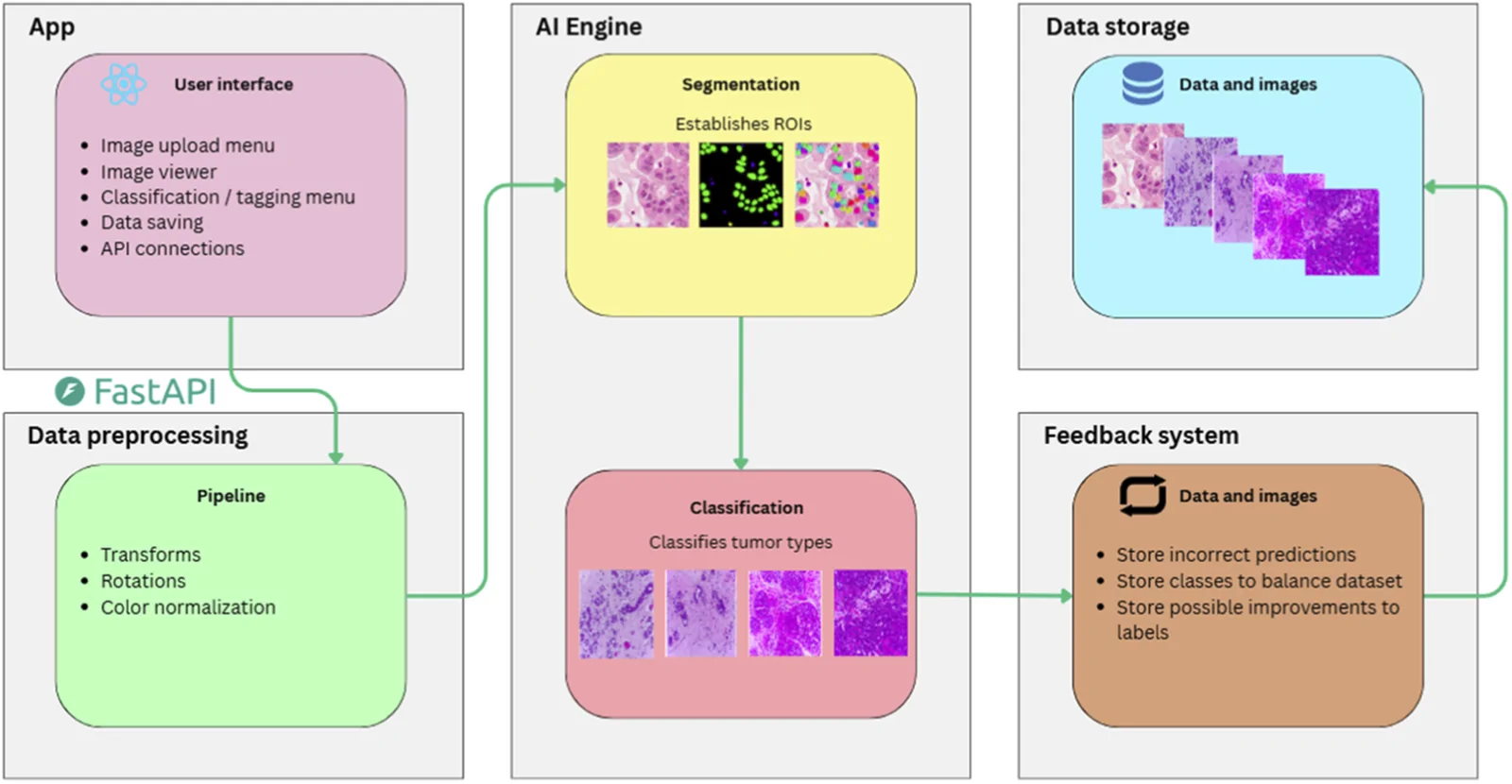
System architecture of HistoBreastCAD, showing the interaction between the React-based frontend, the FastAPI backend, and the PyTorch-based AI engine.

**FIGURE 10:**
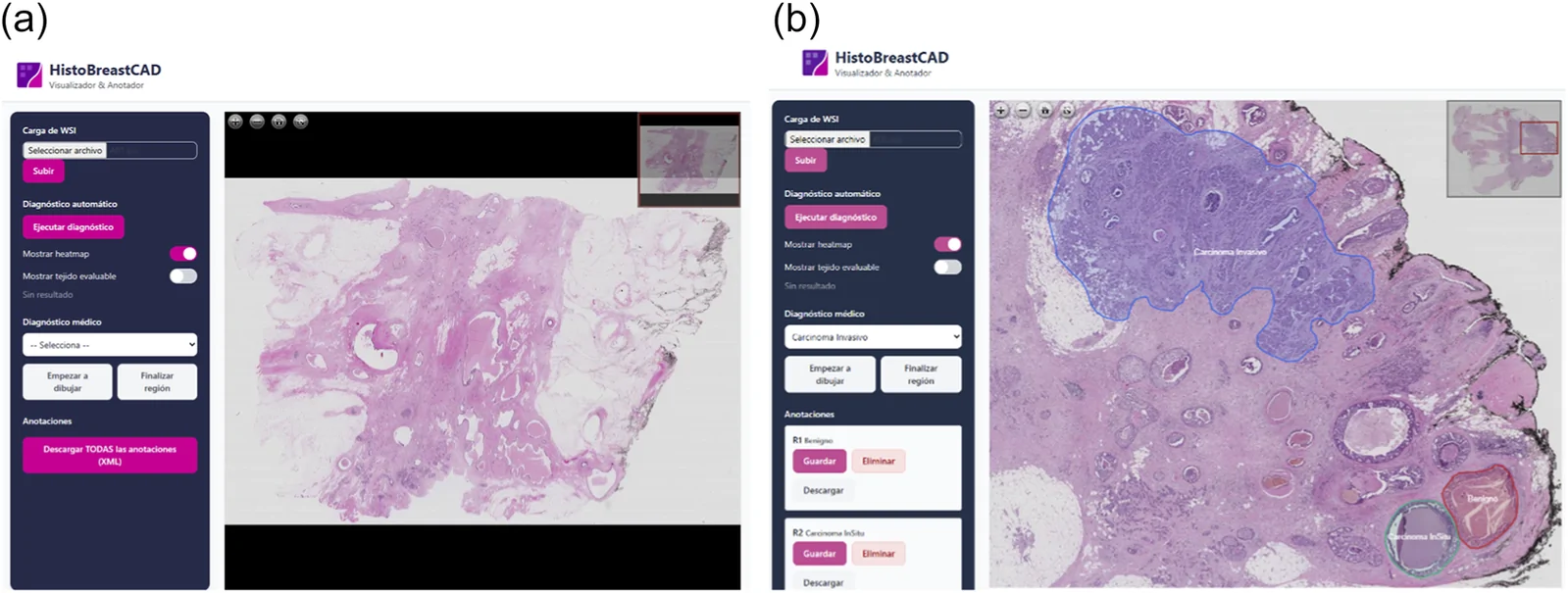
Graphical interface of HistoBreastCAD. **(a)** Main WSI visualization interface, showing the whole-slide viewer and controls for automatic diagnosis, visualization overlays, and manual annotation. **(b)** Example of the manual annotation functionality, in which colored contours represent regions manually delineated and diagnostically labeled by the user/pathologist. These user-defined regions are independent of, and should not be interpreted as, outputs generated by the automatic segmentation or classification models.

## 3 Results

### 3.1 Processed dataset for model training and evaluation

The final segmentation dataset comprised tiles extracted from ten annotated WSIs at two pyramid levels. At L0, preprocessing produced 248,501 tiles, of which 86,020 contained annotated target pixels and 162,481 were negative/background tiles. At L1, 16,421 tiles were available, including 5,462 positive and 10,959 negative tiles. These tile-level categories were used exclusively for training-time balancing; segmentation reference masks and performance evaluation remained pixel based. Five-fold cross-validation was performed at the WSI level, with two complete WSIs used for validation in each fold and no overlap of source-slide identifiers between training and validation. The resulting sampling configuration is summarized in Table 4.

For classification, the CSV-derived dataset contained 19,200 patches originating from 400 labeled source images, with 48 patches extracted from each source image. In each cross-validation fold, 320 source images contributed 15,360 training patches and 80 source images contributed 3,840 validation patches. An additional 10,888 balanced L0 LMDB tiles were incorporated exclusively into the training partition, resulting in 26,248 training samples per fold, with 6,562 samples from each diagnostic class. Validation remained unchanged at 3,840 CSV-derived patches, with 960 patches per class and no LMDB-derived samples. Across the five folds, every one of the 400 source images appeared in validation exactly once, and no source-image overlap was detected between training and validation. The corresponding classification sample distribution is reported in Table 5.

### 3.2 Segmentation performance

Segmentation performance was assessed using pooled pixel-level Precision, Recall, IoU, and micro-Dice under leakage-safe five-fold WSI-grouped cross-validation. As summarized in Table 6, DeepLabV3+ achieved the highest pooled out-of-fold (OOF) micro-Dice among the three standardized semantic segmentation architectures at both pyramid levels. At L1, DeepLabV3+ achieved an OOF micro-Dice of 0.8545 and IoU of 0.7459, compared with 0.8457 and 0.7326 for DeepLabV3 and 0.7582 and 0.6106 for U-Net, respectively. At L0, the corresponding OOF micro-Dice values were 0.8390 for DeepLabV3+, 0.8269 for DeepLabV3, and 0.7701 for U-Net. These results preserved the same pooled performance ordering at both independently evaluated pyramid levels.

Cross-validation also revealed substantial between-fold variability that was not apparent from the pooled OOF estimates alone. At L1, fold-level micro-Dice was 0.7653 ± 0.1721 for DeepLabV3+, 0.7668 ± 0.1579 for DeepLabV3, and 0.6038 ± 0.2812 for U-Net. At L0, the corresponding values were 0.7812 ± 0.1415, 0.7640 ± 0.1271, and 0.6088 ± 0.3204. Thus, although DeepLabV3+ and DeepLabV3 produced similar aggregate performance, U-Net showed markedly greater instability and lower recall in several validation folds. The fold-level distributions are shown in Figure 5.

Because the standardized architecture comparison used checkpoints originally selected with tile-averaged validation Dice, DeepLabV3+ at L0 was subsequently re-trained using pooled pixel-level micro-Dice directly as the checkpoint-selection criterion. This final run achieved an OOF micro-Dice of **0.8390**, IoU of **0.7226**, Precision of **0.8278**, and Recall of **0.8505**, with a fold-level micro-Dice of **0.7967 ± 0.1097**. The aggregate Dice was essentially unchanged relative to the standardized DeepLabV3+ L0 run, whereas Recall increased from 0.7828 to 0.8505 and Precision decreased from 0.9039 to 0.8278. Thus, micro-Dice-based checkpoint selection primarily shifted the operating balance toward greater sensitivity rather than increasing the overall overlap score.

Although the pooled DeepLabV3+ micro-Dice was nominally higher at L1 than at L0, the L0 and L1 experiments used different WSI fold assignments and therefore do not constitute a paired comparison of magnification levels. No claim of statistical superiority between L0 and L1 is made. The L0 configuration was retained for the final WSI workflow to preserve native-resolution spatial detail during patch-wise inference and visualization.

Representative WSI-level segmentation outputs from the evaluated architectures are shown in Figure 4, including the DeepLabV3+ spatial segmentation-probability visualization used in the final workflow.

### 3.3 Classification performance

Classification performance was evaluated using accuracy and macro-averaged F1-score under source-image-grouped five-fold cross-validation, with macro-averaged F1-score used as the primary summary metric. As shown in Table 8, EfficientNetB0 achieved the highest mean macro-F1 among the individual architectures, reaching **0.8142 ± 0.0388** across the five folds. ResNet18-GAP-attention and ResNet18 obtained similar performance, with macro-F1 values of **0.7896 ± 0.0252** and **0.7836 ± 0.0330**, respectively, whereas DarkNet19-GAP-attention showed the lowest performance at **0.7389 ± 0.0303**. EfficientNetB0 outperformed each of the other individual architectures in all five validation folds.

The cross-validation analysis also showed that the apparent advantage of ResNet18-GAP-attention over standard ResNet18 observed in the original development split was not stable across partitions. Their mean macro-F1 difference was only **0.0061**, with ResNet18-GAP-attention performing better in four of five folds, and the paired comparison did not indicate a measurable difference (paired t-test, p = 0.2835). Thus, the attention branch did not provide a reproducible standalone improvement over ResNet18 under the present evaluation protocol.

The frozen EfficientNetB0 + ResNet18-GAP-attention ensemble achieved the highest overall performance, with an accuracy of **0.8283 ± 0.0320** and a macro-F1 of **0.8282 ± 0.0316**. Relative to EfficientNetB0 alone, the ensemble improved accuracy by an average of **0.0140 (95% CI: 0.0053–0.0227)** and macro-F1 **by 0.0141 (95% CI: 0.0047–0.0233)**, outperforming the individual model in all five folds for both metrics. For macro-F1, the paired comparison supported this improvement (paired t-test, p = 0.0134). The ensemble also showed lower between-fold variability than EfficientNetB0 alone (**macro-F1 SD: 0.0316 vs. 0.0388**). The magnitude of this gain was consistent with that observed on the original development split, where the ensemble improved macro-F1 by approximately 0.014. Fold-level performance is illustrated in Figure 6.

Class-specific F1-scores showed a consistent pattern across models. The ensemble obtained the highest mean F1-score in all four diagnostic categories: **0.8151** for normal tissue, **0.8070** for benign lesions, **0.8146** for carcinoma *in situ*, and **0.8762** for invasive carcinoma. Invasive carcinoma was the best-performing category across all evaluated architectures, whereas benign lesions showed the lowest average F1-score. These results indicate that the classification difficulty was not uniformly distributed across diagnostic categories.

Importantly, these cross-validation estimates quantify the performance of the patch-classification module on held-out BACH source images. Validation contained only CSV-derived patches and no LMDB-derived WSI tiles; therefore, these results should not be interpreted as an estimate of end-to-end WSI-level diagnostic performance of the complete HistoBreastCAD pipeline.

### 3.4 Exploratory reader concordance on the BACH test set

The exploratory reader study included the 100 microscopy images from the held-out BACH test set, for which model predictions were available in all cases. Reader 1 provided 96 valid four-class annotations and Reader 2 provided 99, while 95 images had valid paired annotations from both readers. The diagnostic distributions of the readers and the evaluated models are summarized in Table 9. Model-to-reader concordance was calculated using the valid cases available for each reader, whereas direct interobserver agreement was restricted to the 95 paired cases.

Direct comparison between the two readers showed observed agreement of **66.32%** across the 95 paired images, corresponding to a Cohen’s κ of **0.5105** and a quadratic-weighted κ of **0.6161**. The corresponding confusion matrix is shown in Figure 7a. Although the diagonal predominated, residual disagreement remained concentrated in selected diagnostic transitions, including ten cases classified as invasive carcinoma by Reader 1 and benign lesion by Reader 2.

Against Reader 1, EfficientNetB0 achieved the highest unweighted model-to-reader concordance, with Cohen’s κ = **0.3242** and quadratic-weighted κ = **0.6709** (n = 96). The frozen EfficientNetB0 + ResNet18-GAP-attention ensemble produced a very similar result, with κ = **0.3183** and weighted κ = **0.6578**, while ResNet18-GAP-attention alone reached κ = **0.2493** and weighted κ = **0.5636**. EfficientNetB0 and the ensemble both showed an observed agreement of **47.92%** with Reader 1. The ensemble-versus-Reader 1 confusion matrix is presented in Figure 7b.

Concordance was lower against Reader 2. EfficientNetB0 obtained Cohen’s κ = **0.2183** and weighted κ = **0.4799** (n = 99), whereas the ensemble reached κ = **0.2125** and weighted κ = **0.4514**. ResNet18-GAP-attention obtained κ = **0.1748** and weighted κ = **0.4339**. Thus, all three models showed greater concordance with Reader 1 than with Reader 2, while remaining below the direct reader-to-reader agreement.

Notably, the improvement obtained by the ensemble during source-image-grouped cross-validation did not translate into higher concordance with either reader. Relative to EfficientNetB0 alone, the ensemble changed Cohen’s κ by only **−0.0059** against Reader 1 and **−0.0058** against Reader 2. These differences are small and should not be interpreted as evidence that the ensemble performs worse in the reader study; rather, the benchmark improvement observed during internal classification evaluation did not produce a corresponding increase in reader concordance in the held-out BACH test material.

A consistent distributional difference was also observed between the model predictions and the reader labels. The ensemble and EfficientNetB0 classified 35 and 36 of the 100 images as normal tissue, respectively, whereas each reader assigned the normal category to only 10 cases. Conversely, Reader 2 assigned a larger proportion of images to the benign category (53/99) than either model. These differences contributed substantially to the observed model-to-reader disagreement and suggest that the classification behavior learned from the development data did not transfer uniformly to the reader-study material.

Taken together, these results indicate that the exploratory reader study should be interpreted as a concordance assessment rather than as an estimate of clinical diagnostic accuracy. The two readers showed higher agreement with each other than any model achieved with either reader, and no consensus or adjudicated reference diagnosis was available to determine correctness in discordant cases. Accordingly, the reader-study results complement the internal classification evaluation but do not establish clinical performance of the HistoBreastCAD system.

### 3.5 Integrated system results and visualization

The final HistoBreastCAD framework integrates segmentation, classification, and interactive visualization within a single digital pathology workflow. The internal inference logic of the AI engine is shown in Figure 8, whereas the complete client-server system architecture is presented in Figure 9. These figures show how the segmentation output is used to guide the classification stage and how both outputs are subsequently delivered to the graphical interface for clinical review.

Figure 10 illustrates the user-facing manual annotation functionality of the interface. The colored regions shown in this figure correspond to annotations manually delineated and labeled by the user and should not be interpreted as automatic segmentation masks or model-generated classification regions. This functionality enables pathologists to record, correct, or complement regions during WSI review. Automated model outputs are displayed through dedicated tissue-detection and spatial classification-probability overlays, which are independent from the manual annotation layer illustrated in Figure 10.

Overall, these results demonstrate the operational integration of tissue-region segmentation, multiclass classification, spatial visualization of model predictions, and interactive WSI review within the HistoBreastCAD environment. Quantitative performance of the segmentation and classification modules was evaluated separately, as reported in Sections 3.2 and 3.3, and should not be interpreted as a single end-to-end WSI diagnostic-performance estimate. The interface results demonstrate the implementation of the analytical workflow and its visualization capabilities, while Figure 10 specifically illustrates manual annotation functionality rather than formal model explainability.

## 4 Discussion

The findings of this study support the feasibility of implementing deep learning–based segmentation, multiclass classification, and visual decision support as interconnected components of a breast histopathology CAD workflow. As discussed above, sequential ROI localization and classification have previously been explored in integrated WSI analysis systems (Gecer et al., 2018; Wu et al., 2020; Hsu et al., 2024). Therefore, the contribution of HistoBreastCAD should not be interpreted as the introduction of a new segmentation–classification paradigm or a novel neural-network architecture. Instead, the present work evaluates a specific implementation of this established paradigm in which tissue-region segmentation guides subsequent four-class histopathological classification and the resulting spatial predictions are delivered within an interactive WSI visualization environment. From a healthcare analytics perspective, this system-level integration is relevant because CAD tools can provide value not only on predictive performance but also through the organization of computational outputs for image review and diagnostic decision support (Guo et al., 2022; Campanella et al., 2019).

### 4.1 Segmentation performance and relevance to the integrated workflow

The cross-validated segmentation analysis provides a more informative estimate of model behavior than pooled performance values alone. DeepLabV3+ achieved the highest pooled OOF micro-Dice among the three standardized semantic segmentation architectures at both evaluated pyramid levels, reaching 0.8545 at L1 and 0.8390 at L0. However, DeepLabV3 produced closely comparable performance (0.8457 at L1 and 0.8269 at L0), indicating that the results do not support a large performance separation between the two DeepLab architectures. U-Net showed lower pooled Dice and substantially greater between-fold variability, particularly in folds in which recall deteriorated. These findings are consistent with the suitability of semantic segmentation architectures for histopathological tissue analysis and with previous work applying multiscale and encoder–decoder approaches to breast histopathology segmentation (Chen et al., 2018; Priego-Torres et al., 2020; Drioua et al., 2023; Graham et al., 2019; Chen et al., 2022).

An important finding of the WSI-grouped cross-validation was the magnitude of between-fold and between-slide variability. The pooled OOF values alone concealed substantial differences among validation partitions: for example, the fold-level micro-Dice standard deviation ranged from approximately 0.13–0.17 for the DeepLab models and was markedly larger for U-Net. Moreover, several WSIs were consistently difficult across independent architectures, particularly slides such as a08 and a10 in the L0 evaluation. Because the same difficult slides tended to reduce recall across different models, this pattern suggests that part of the observed variability reflects slide-specific characteristics rather than architecture-specific failure alone. Possible contributors include differences in tissue morphology, staining, image quality, or annotation complexity, although these factors were not separately quantified in the present study.

The re-analysis also demonstrated that the choice of metric aggregation can materially affect checkpoint selection. Tile-averaged Dice was found to be insensitive to poor predictions on positive tissue when large numbers of empty-background tiles were present, which could yield apparently high validation scores for checkpoints with very low recall. Re-training DeepLabV3+ at L0 with pooled pixel-level micro-Dice as the selection criterion removed this failure mode without artificially increasing the final overlap metric: OOF micro-Dice remained 0.8390, while Recall increased from 0.7828 to 0.8505 and Precision decreased from 0.9039 to 0.8278. This result indicates that the micro-Dice-based selection criterion primarily changed the precision–recall operating balance rather than producing a higher aggregate Dice score. Because the checkpoints used in the standardized architecture comparison had originally been selected using tile-averaged Dice, the relative differences among architectures should be interpreted descriptively. A fully controlled comparison under micro-Dice-based checkpoint selection would require re-training all architectures using the same selection criterion.

Within HistoBreastCAD, segmentation is intended primarily as an upstream tissue-selection component rather than as an independent diagnostic endpoint. By separating annotated tissue of interest from non-target/background regions, the segmentation stage provides spatial guidance for subsequent patch-based classification and WSI visualization. The L0 DeepLabV3+ configuration was retained in the final workflow to preserve native-resolution spatial information during patch-wise inference, rather than because L0 was statistically superior to L1; the two pyramid-level experiments used different fold compositions and therefore were not treated as a paired resolution comparison. Importantly, the segmentation results characterize the segmentation module itself and should not be interpreted as an end-to-end measure of diagnostic performance of the complete HistoBreastCAD pipeline.

### 4.2 Classification performance and ensemble behavior

The source-image-grouped cross-validation results provide a more robust estimate of classification performance than the original single development split. EfficientNetB0 was the strongest individual architecture, achieving a mean macro-F1 of **0.8142 ± 0.0388** and outperforming each of the other individual models in all five validation folds. ResNet18-GAP-attention and standard ResNet18 achieved similar mean macro-F1 values of **0.7896 ± 0.0252** and **0.7836 ± 0.0330**, respectively, whereas DarkNet19-GAP-attention showed the lowest performance (**0.7389 ± 0.0303**). The small difference between the two ResNet18 configurations was not statistically distinguishable (paired t-test, p = 0.2835), indicating that the GAP-attention branch did not provide a reproducible standalone improvement under the present evaluation protocol.

Despite the limited standalone difference between the two ResNet18 configurations, the frozen EfficientNetB0 + ResNet18-GAP-attention ensemble produced a consistent improvement over its individual members. The ensemble achieved a mean macro-F1 of **0.8282 ± 0.0316**, exceeding EfficientNetB0 in all five folds by an average of **0.0141 ± 0.0074** (paired t-test, p = 0.0134). It also showed lower between-fold variability than EfficientNetB0 alone (SD 0.0316 versus 0.0388). Importantly, this ensemble configuration had been selected on the fixed development split before cross-validation and was not re-selected using the five-fold results. The repeated gain across all five partitions therefore supports the stability of the probability-averaging strategy within the evaluated BACH classification domain.

These results are consistent with previous breast histopathology studies showing that convolutional backbones and ensemble strategies can provide robust multiclass representations when combined with transfer learning and probability-level fusion (Balasubramanian et al., 2024; Hameed et al., 2020; Hameed et al., 2022; Srikantamurthy et al., 2023; Spanhol et al., 2016). EfficientNetB0 combines compound scaling of network depth, width, and resolution (Tan and Le, 2019), whereas the ResNet family relies on residual learning to facilitate optimization (He et al., 2016). However, the present experiments do not establish which architectural properties caused the ensemble gain. Rather, the empirical finding is that averaging the probability distributions of EfficientNetB0 and ResNet18-GAP-attention reduced fold-dependent error sufficiently to improve macro-F1 consistently. The ensemble also achieved the highest mean F1-score in each diagnostic class, with invasive carcinoma showing the strongest performance and benign lesions the lowest average F1-score across the evaluated models.

A relevant distinction emerged when the benchmark and reader-study results were considered together. Although the ensemble improved macro-F1 consistently across all five BACH validation folds, this advantage did not translate into greater concordance with either pathologist in the held-out BACH test set. EfficientNetB0 and the ensemble obtained very similar Cohen’s κ values against Reader 1 (0.3242 and 0.3183) and Reader 2 (0.2183 and 0.2125), respectively. The small decreases observed for the ensemble should not be interpreted as evidence of inferior clinical performance; rather, they indicate that the reproducible gain observed within the BACH validation domain did not produce a corresponding increase in reader concordance. This contrast highlights the distinction between internal predictive performance and agreement on a separate held-out BACH image set evaluated through reader annotations and reinforces the need for independent external evaluation before broader claims of clinical generalizability can be supported. Moreover, because the cross-validation validation sets contained only BACH-derived CSV patches while the additional LMDB WSI tiles contributed exclusively to training, the observed between-fold variability primarily reflects sensitivity to the partitioning of the BACH source images rather than variability across independent WSIs.

### 4.3 Comparison with recent BACH-based methods

To contextualize the performance and methodological scope of HistoBreastCAD, Table 11 compares the proposed framework with representative recent approaches evaluated on the BACH dataset. Because the included studies differ in task definition, dataset partitioning, augmentation strategies, and evaluation protocols, the reported values should be interpreted as literature-based benchmarks rather than as results from a direct head-to-head experimental comparison. Metrics that were not reported or were not applicable to the task evaluated in the original studies are indicated as NR rather than inferred.

**TABLE 11:** Comparison of recent BACH-based breast histopathology approaches and HistoBreastCAD.

Study	BACH task	Main approach	Dice	IoU	Accuracy	F1-score	Main strengths	Main limitations
Yılmaz et al. (Yilmaz et al., 2025)	4-class microscopy-image classification	MViTv2-Base; broad CNN/ViT benchmark	NR	NR	0.9167	0.9159	Systematic comparison of nearly 30 CNN and ViT architectures; evaluates transformer-based representation	Classification only; no WSI segmentation or integrated CAD workflow
Ogundokun et al. (Ogundokun et al., 2025)	4-class image classification	ResNet50 deep features + optimized ML classifiers + soft-voting ensemble	NR	NR	≈0.900	≈0.863	Ensemble fusion with optimization; combines complementary ML classifiers	Classification only; no WSI segmentation or interactive WSI analysis
Jia et al. (Jia et al., 2025), DenLsNet-C	4-class microscopy-image classification	DenseNet + LSTM hybrid	NR	NR	0.9375	0.9378	Combines spatial CNN features with sequential feature modeling; strong multiclass performance	Classification only; random 80:20 split; no WSI-level segmentation or integrated visualization
Zhou et al. (Zhou et al., 2025), ResViT-GANNet	4-class microscopy-image classification	Parallel ResNet50 + ViT, TAMA attention fusion, StyleGAN2-ADA	NR	NR	0.9640	0.9635	CNN–Transformer local/global feature integration; generative augmentation; Grad-CAM; statistical evaluation	Classification only; no WSI segmentation; synthetic augmentation complicates direct comparison
Inamdar and Shaila (Inamdar and Shaila, 2026)	Segmentation + benign/malignant classification	MobileNetV2 + U-Net + label propagation	0.934 ± 0.008	0.876 ± 0.010	0.940 ± 0.008	0.929 ± 0.008	Segmentation and classification; lightweight architecture; repeated experiments; cross-dataset validation; XAI	Classification task is binary rather than BACH four-class categorization; not an integrated four-class WSI review system
HistoBreastCAD (proposed)	WSI tissue-region segmentation + 4-class patch classification	DeepLabV3+ segmentation → EfficientNetB0 + ResNet18-GAP-attn equal-weight soft voting → spatial WSI visualization	0.8390*	0.7226*	0.8283 ± 0.0320†	0.8282 ± 0.0316†	Leakage-safe source-level evaluation; WSI-grouped segmentation CV; source-image-grouped classification CV; integrated segmentation, four-class classification, spatial mapping, and interactive WSI review	Single public benchmark for internal evaluation; no independent WSI-level end-to-end performance estimate; limited exploratory reader study; established CNN architectures; external multi-institutional validation still required

NR, not reported or not applicable in the original study. *HistoBreastCAD, segmentation values correspond to pooled OOF, pixel-level metrics from the final DeepLabV3+ L0 five-fold WSI-grouped evaluation. HistoBreastCAD, classification Accuracy and macro-F1 correspond to mean ± SD, from source-image-grouped five-fold cross-validation of the frozen EfficientNetB0 + ResNet18-GAP-attention ensemble. Metrics across studies were obtained under different experimental protocols and should not be interpreted as a direct ranking.

As shown in Table 11, recent BACH-based studies report strong performance using WSI-oriented methods, conventional CNNs, ensembles, hybrid CNN–Transformer architectures, and segmentation–classification strategies. Several classification-focused approaches report higher numerical classification metrics than those obtained by HistoBreastCAD. However, direct numerical ranking is not appropriate because the evaluated tasks and validation protocols differ substantially; some studies address binary classification, whereas others address four-class microscopy-image classification or combined segmentation and classification tasks. The cross-validated HistoBreastCAD results should therefore be interpreted within the specific source-separated evaluation protocol used in the present study rather than as evidence of superiority over the listed methods.

### 4.4 Reader concordance and interobserver variability

The exploratory reader study provides a complementary perspective on the internal classification results by examining agreement under an independent interpretation setting. Direct interobserver agreement was higher than model-to-reader concordance: the two readers achieved an observed agreement of **66.32%**, Cohen’s κ = **0.5105**, and quadratic-weighted κ = **0.6161** across the 95 images with valid paired annotations. In comparison, EfficientNetB0 achieved Cohen’s κ values of **0.3242** against Reader 1 and **0.2183** against Reader 2, while the corresponding values for the frozen ensemble were **0.3183** and **0.2125**. Thus, the models showed measurable agreement with both readers, but remained below the level of agreement observed between the readers themselves.

This difference between internal classification performance and reader concordance should not be interpreted as contradictory. The cross-validation results quantify predictive performance relative to the BACH reference labels under source-image-separated validation, whereas the reader study measures agreement with individual pathological interpretations on the held-out BACH test images. These are distinct evaluation questions. In breast histopathology, diagnostic boundaries among benign lesions, carcinoma *in situ*, and invasive carcinoma can involve subtle morphological distinctions and may be interpreted differently by individual specialists (Rashmi et al., 2022; Guo et al., 2022; Harrison et al., 2023). Accordingly, concordance with a reader is not equivalent to classification accuracy against an adjudicated reference standard.

The interobserver confusion pattern further illustrates this limitation. Although agreement was moderate overall, ten images classified as invasive carcinoma by Reader 1 were categorized as benign by Reader 2, indicating that clinically relevant disagreement remained even after the majority of cases were aligned between readers. This variability prevents either reader from being treated as an unequivocal ground truth in the present material. The reader annotations should therefore be interpreted as independent expert opinions used for concordance analysis rather than as definitive labels against which clinical diagnostic accuracy can be established.

A particularly informative finding was that the ensemble gain observed during BACH cross-validation did not translate into increased reader concordance. The EfficientNetB0 + ResNet18-GAP-attention ensemble improved macro-F1 over EfficientNetB0 in all five internal validation folds, with a mean gain of approximately **0.014**, yet Cohen’s κ was slightly lower for the ensemble against both Reader 1 (0.3183 versus 0.3242) and Reader 2 (0.2125 versus 0.2183). These differences in κ are small and should not be interpreted as evidence that the ensemble is clinically inferior. Rather, they show that a reproducible improvement within the benchmark domain does not necessarily produce a corresponding improvement in agreement under a held-out test-image and reader-annotation setting.

The prediction distributions also differed noticeably from those of the readers. EfficientNetB0 and the ensemble assigned approximately 35% of the reader-study images to the normal category, whereas each reader used this category for only 10 cases. Reader 2, in contrast, assigned 53 of 99 images to the benign category. Such distributional differences may reflect differences between the label distributions learned from the labeled BACH development set and the interpretations assigned by the two readers to the BACH test images. Because the corresponding test-set reference labels were unavailable in the present study, the source of these differences cannot be determined.

These findings reinforce the interpretation of HistoBreastCAD as a proof-of-concept decision-support framework rather than as an autonomous diagnostic system. The reader study demonstrates that benchmark performance, model-to-reader agreement, and reader-to-reader agreement capture different properties of the evaluation setting. Larger prospective multi-reader studies with predefined reading protocols, complete case assessment, and consensus or adjudicated reference diagnoses will be required to determine whether the system provides clinically meaningful diagnostic support.

### 4.5 Value of integration for digital pathology workflows

A central contribution of this study lies in the integration of segmentation, multiclass classification, spatial visualization, and interactive WSI review within a single CAD environment. Previous studies have demonstrated ROI localization, segmentation, and diagnostic classification either as individual tasks or as components of integrated histopathology pipelines (Guo et al., 2022; Labrada and Barkana, 2023; Campanella et al., 2019). HistoBreastCAD therefore does not introduce a new segmentation–classification paradigm; rather, it implements these established analytical components as connected stages of a BACH-based workflow. As illustrated in Figures 8, 9, segmentation-derived tissue information, patch-level classification, spatial mapping of model outputs, and the web-based review interface are connected within the same inference and visualization architecture.

This integration has several practical implications for digital pathology analysis. First, the segmentation stage provides a spatial mechanism for identifying tissue regions that can subsequently be considered by the classification module, thereby limiting detailed analysis to selected regions rather than treating the complete gigapixel slide uniformly. Second, patch-level classification probabilities can be mapped back to their spatial locations on the original WSI, allowing predictions to be reviewed in their histological context rather than as isolated numerical outputs. Third, the web-based interface combines these computational outputs with WSI pan, zoom, and manual annotation functionality, providing an operational link between model inference and user review. These features distinguish the implemented framework from evaluation of a standalone patch classifier alone.

The user-facing component of the integrated workflow is illustrated in Figure 10. The regions displayed in this figure correspond to manual annotations created within the interface and are distinct from the automated tissue-detection and spatial classification-probability overlays generated by the analytical pipeline. Together, these layers allow users to inspect model-derived spatial information while independently recording or modifying annotations during WSI review. This separation between automated predictions and user-generated annotations is important for positioning HistoBreastCAD as a decision-support environment in which computational outputs remain available for human inspection rather than being presented as autonomous diagnostic decisions.

### 4.6 Dataset limitations and methodological constraints

A major limitation of the present study is that model development and quantitative internal performance evaluation relied primarily on the BACH dataset (Aresta et al., 2019). Although BACH provides a well-established benchmark for breast histopathology research, a single benchmark cannot represent the full variability encountered across institutions, staining protocols, tissue-processing procedures, scanner platforms, and patient populations. Consequently, the cross-validated segmentation and classification results should be interpreted as evidence of feasibility within the evaluated experimental setting rather than as evidence of external clinical generalizability. The held-out BACH test set provided an additional reader-concordance assessment, but it remained part of the same benchmark source and did not constitute an independent external cohort. Moreover, because the corresponding reference labels were not available to the present study, it could not be used to estimate conventional test-set diagnostic accuracy. Validation on heterogeneous multi-institutional cohorts is therefore required before broader clinical applicability can be established.

A second limitation concerns the composition and variability of the segmentation data. The segmentation experiments were based on only ten annotated WSIs, and the available tiles were imbalanced between positive and negative/background regions. Training-time 1:1 sampling reduced the influence of this imbalance within each fold, but it does not increase the underlying diversity of the source WSIs. WSI-grouped cross-validation revealed substantial between-fold and between-slide variability, indicating that performance remains sensitive to the characteristics of the held-out slides. In addition, only the final DeepLabV3+ L0 configuration was re-trained using micro-Dice directly for checkpoint selection; the checkpoints used for the standardized architecture comparison had originally been selected using tile-averaged Dice and were subsequently re-evaluated using pooled pixel-level metrics. Therefore, differences among segmentation architectures should be interpreted descriptively rather than as a fully controlled comparison under identical checkpoint-selection criteria.

The classification design also has source-specific limitations. The additional LMDB-derived WSI tiles contributed exclusively to training, whereas validation in every fold consisted entirely of held-out BACH source-image patches. Accordingly, the reported cross-validation variability quantifies sensitivity to different partitions of the 400 BACH source images and does not estimate variability across independent WSIs. Furthermore, the assumed disjointness between the CSV-derived BACH material and the WSI-derived LMDB material is based on dataset provenance rather than on a patient- or specimen-level identifier shared by both sources. Although no source-image overlap occurred within the BACH folds and no LMDB sample entered validation, a common identifier system was unavailable to independently verify specimen-level disjointness across the two data sources.

The exploratory reader study is subject to additional limitations. Although the 100 BACH test images were presented to both readers, valid four-class annotations were available for 96 images from Reader 1 and 99 from Reader 2, with 95 images having paired valid annotations. Only two readers participated, a single reading round was performed, and no third-reader adjudication or consensus reference diagnosis was available. The observed interobserver agreement was moderate rather than complete, and clinically relevant disagreements remained between the readers. Consequently, model-to-reader concordance cannot be interpreted as diagnostic accuracy against clinical ground truth. Larger prospectively designed multi-reader studies with complete case assessment and consensus or adjudicated reference standards are required to evaluate clinical performance more rigorously.

Finally, the individual analytical components were quantitatively evaluated separately rather than through a single end-to-end WSI diagnostic metric. Segmentation metrics quantify pixel-level tissue-region delineation, whereas classification metrics quantify patch-level predictions on held-out BACH source images. The integrated interface demonstrates that these components can be connected operationally, but the present study does not establish a single WSI-level diagnostic accuracy for the complete segmentation–classification pipeline. Similarly, the spatial classification-probability overlay was designed for visualization and was not evaluated as a formal explainability method using localization or attribution metrics. Future studies should therefore combine external WSI-level validation with formal explainability assessment and prospective workflow evaluation.

### 4.7 Implications for healthcare analytics and future work

From a healthcare analytics perspective, the contribution of HistoBreastCAD lies in combining predictive modeling with an implemented pathology-analysis workflow. The system structures WSI analysis into sequential tissue-region selection, multiclass categorization, spatial mapping of model outputs, and interactive review. In this context, the analytical contribution extends beyond isolated model performance by organizing computational outputs in a form that can be inspected within the original histopathological image. This positioning is consistent with the broader role of CAD systems as decision-support tools intended to assist, rather than replace, specialist review (Guo et al., 2022; Campanella et al., 2019).

Future work should first prioritize external validation using independent, multi-institutional cohorts acquired under different staining protocols, tissue-processing conditions, and scanner platforms. Such evaluation is necessary to determine whether the cross-validated performance observed within the BACH domain generalizes to broader WSI populations. Future experimental designs should also include held-out WSI material in classification validation so that variability associated with the WSI-derived training domain can be quantified directly.

Second, future work should incorporate formal explainability and localization analyses. The current spatial classification-probability overlay was implemented as a visualization mechanism and was not evaluated as a formal explainability method. Future studies could therefore incorporate class-activation or attribution-based approaches and assess their spatial correspondence with expert-defined regions using quantitative localization metrics. This would allow interpretability claims to be evaluated independently from predictive performance rather than inferred from qualitative visualization alone.

Third, clinical evaluation should be implemented through prospectively designed multi-reader studies with predefined reading protocols, complete case assessment, and consensus or adjudicated reference diagnoses. Such studies could additionally evaluate workflow-oriented outcomes such as review time, annotation burden, diagnostic consistency, and user interaction with automated overlays. These operational endpoints were not measured in the present study and should therefore be assessed directly before claims regarding workflow efficiency or clinical utility are made.

## 5 Conclusion

This study presented HistoBreastCAD, an integrated deep learning–based computer-aided diagnosis framework for breast histopathology analysis in whole-slide images. The system combines semantic segmentation, multiclass classification, spatial mapping of model outputs, and web-based WSI interaction within a unified analytical workflow. Building on previously established segmentation and classification approaches, the contribution of HistoBreastCAD lies primarily in the implementation and evaluation of these components as interconnected stages of a digital pathology workflow rather than in the introduction of a new neural-network architecture.

Under leakage-safe WSI-grouped five-fold cross-validation, the final DeepLabV3+ L0 segmentation configuration achieved an OOF micro-Dice of **0.8390**, IoU of **0.7226**, Precision of **0.8278**, and Recall of **0.8505**. For classification, source-image-grouped five-fold cross-validation identified EfficientNetB0 as the strongest individual backbone, while the frozen EfficientNetB0 + ResNet18-GAP-attention ensemble achieved the highest overall performance, with an Accuracy of **0.8283 ± 0.0320** and macro-F1 of **0.8282 ± 0.0316**. The ensemble exceeded EfficientNetB0 in all five classification folds, demonstrating that its modest improvement was reproducible across the evaluated BACH partitions.

The exploratory reader study on the held-out BACH test images provided a complementary but distinct assessment. Interobserver agreement between the two pathologists was higher than model-to-reader concordance (Cohen’s κ = **0.5105**, n = 95). EfficientNetB0 achieved κ values of **0.3242** and **0.2183** against Readers 1 and 2, respectively, while the corresponding ensemble values were **0.3183** and **0.2125**. Thus, the ensemble improvement observed during internal cross-validation did not translate into increased reader concordance. Because no consensus or adjudicated diagnostic reference standard was available, these findings should be interpreted as exploratory concordance rather than as an estimate of clinical diagnostic accuracy.

Beyond individual model performance, the study demonstrates the operational integration of tissue-region segmentation, patch-level classification, spatial visualization, and manual WSI review within a single environment. However, segmentation and classification were quantitatively evaluated as separate analytical components, and no single end-to-end WSI-level diagnostic accuracy was established for the complete pipeline. Likewise, the spatial classification-probability overlay provides a visualization of model predictions but was not evaluated as a formal explainability method.

The present findings should therefore be regarded as proof-of-concept evidence within a predominantly BACH-based experimental setting. External validation using independent, heterogeneous, multi-institutional WSI cohorts, prospective multi-reader studies with adjudicated reference diagnoses, and formal quantitative assessment of localization and explainability will be necessary before broader claims of clinical generalizability or utility can be supported. Within these limitations, HistoBreastCAD demonstrates the feasibility of integrating established deep learning components into a source-separated, WSI-oriented analytical and review workflow for breast histopathology.

## Data Availability

The data supporting this study are available in Mendeley Data (Celi Vega et al., 2026) (doi: 10.17632/86rg2yg5ms.2).
