# Supplementary material for: HistoBreastCAD: an integrated deep learning framework for segmentation and multiclass classification of whole-slide breast histopathology images
Source: Front Bioinform. 2026 Oct 13;6:1870283. doi: 10.3389/fbinf.2026.1870283 (PMC13710403; doi:10.3389/fbinf.2026.1870283)
Supplement: Supplementary file 1 [file Supplementaryfile1.docx]

Supplementary Material

# Annex A
